# Characterization and Application of Pr–Co Codoped BiFeO_3_ for Cr(VI) Reduction in the Process of Visible-Light Photocatalysis

**DOI:** 10.1021/acsomega.6c00081

**Published:** 2026-06-25

**Authors:** Aarti Gupta, Rim Zgueb, Tina Škorjanc, Mohit Sahni, Barbara Ressel, Dorota Korte

**Affiliations:** † Laboratory for Environmental & Life Sciences, 119110 University of Nova Gorica, Vipavska 13, Nova Gorica 5000, Slovenia; ‡ Materials Research Laboratory, University of Nova Gorica, Vipavska 11c, Ajdovščina 5270, Slovenia; § Advanced Material Research Laboratory, Department of Physics and Environmental Sciences, 365068 Sharda University, Greater Noida, Uttar Pradesh 201310, India; ∥ Laboratory of Quantum Optics, University of Nova Gorica, Vipavska 11c, Ajdovščina 5270, Slovenia

## Abstract

The aim of the study was to apply a combined photothermal spectrometer that integrates BDS and PPE systems for multiparameter characterization of Pr–Co codoped BFO photocatalysts within a single measurement. Such a system provided information on the material’s thermal diffusivity, conductivity, band gap, and carrier lifetime. The results indicate that moderate doping improves thermal transport and charge carrier separation, while excessive doping introduces additional recombination centers that accelerate charge carrier recombination and reduce heat propagation within the material. The photothermal measurements were supported by structural analysis of the samples performed by Powder X-ray Diffraction technique, Scanning Electron Microscopy coupled with Energy Dispersive X-ray Analysis, Fourier Transform Infrared Spectroscopy, and X-ray Photoelectron Spectroscopy. It was found that Pr­(III) and Co­(III) substituted into the BFO lattice without the formation of secondary phases and were accompanied by the reduction of crystallite size and morphological evolution. The photocatalytic performance of BFO materials was further verified in the reduction process of Cr­(VI) to Cr­(III) under natural environmental conditions using solar sunlight without the use of artificial light sources. This indicates that codoping significantly enhances Cr­(VI) reduction efficiency, achieving ∼96% within 120 min of the photocatalytic process. A correlation between structural, optical, and thermal parameters was also found, providing a basis for designing stable and efficient photocatalysts for environmental applications under solar sunlight.

## Graphical abstract

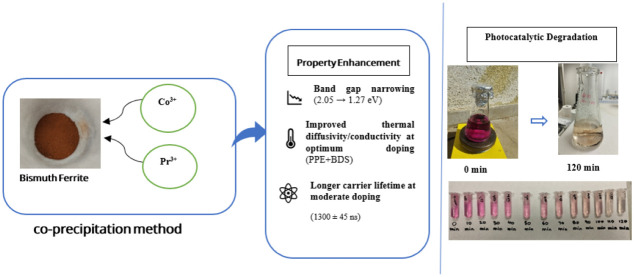

## 1 Introduction

Environmental contamination by heavy metals has become one of the most pressing challenges in modern society.^1–3^ Among them, hexavalent chromium (Cr­(VI)) is of particular concern because of its high toxicity, high solubility, and carcinogenic nature.^4^ Cr­(VI)-containing effluents generated from electroplating, leather tanning, textiles, and mining industries pose serious risks to both ecosystems and human health. Conventional water purification technologies for Cr­(VI) reduction, such as chemical precipitation, adsorption, and membrane separation, suffer from several limitations that include high operational costs, generation of secondary waste, and low efficiency at trace levels of contaminants.^4^ Therefore, advanced approaches capable of selective, efficient, and sustainable reduction of Cr­(VI) to less toxic trivalent chromium (Cr­(III)) are required to be developed.

Photocatalysis is widely used as a green technology for efficient pollutant reduction and/or degradation. In this process, semiconductor materials absorb solar energy and produce electron–hole pairs, which then participate in redox reactions. The photocatalytic processes offer significant economic and environmental advantages, as they can operate under mild conditions and utilize renewable solar energy. When integrated with complementary systems, photocatalysis can reduce operational costs and minimize resource consumption, making it a viable candidate for long-term sustainable and industrial-scale environmental remediation.^5–8^ Nowadays, the most frequently used photocatalyst is titanium dioxide (TiO_2_)^9–13^ because of its high photocatalytic activity, commercial availability at low cost, chemical stability, and nontoxicity. Although TiO_2_ is the most used, it suffers from some limitations such as a large band gap (∼3.2 eV), due to which it only absorbs UV radiation, representing only 4–5% of the solar spectrum. Thus, efforts have been made to develop visible-light-active photocatalysts with narrower band gaps and better photocatalytic performance.^14^ An attention has been given to bismuth ferrite (BiFeO_3_, BFO)^15–19^ that is a multifunctional material. It is cost-effective, environmentally friendly, chemically stable, and nontoxic.^15–18,20^ thus making it a promising material in the field of next-generation electronics and environmental technologies. As a result of its high-performance ability that is defined by its high-density storage potential and fast switching speeds (even down to 20 ns), BFO is used in the fields of spintronics, photovoltaic technology, and as a nonvolatile memory. Since BFO also operates with low energy consumption at the level of a few pJ up to hundreds of fJ, it is applicable in AI and neuromorphic computing.^21–23^ Furthermore, BFO exhibits magnetic, ferroelectric, as well as piezoelectric properties. Thus, it is used as a gas sensor, magnetic field sensor, optical and thermal sensor, and tactile and pressure sensor.^24,25^ Special attention is drawn to BFO’s ability to absorb visible light, which makes it interesting for photocatalytic applications since it does not require the implementation of artificial light sources, as is needed in the case of UV-driven catalysts (e.g., TiO_2_). The magnetic properties of BFO enables its cost-effective recovery from remediated water what makes it reusable.^26,27^ Modification of pure BFO materials can further improve its photocatalytic properties, making it competitive for real-world environmental water treatment processes.

Despite all of these advantages, pure BFO suffers from several intrinsic drawbacks, such as high charge carrier recombination rates and structural instability, which are factors that significantly limit its photocatalytic efficiency. To overcome these limitations, various strategies have been proposed to improve its properties, including surface modification, elemental doping, and composite formation. Among them, codoping with rare-earth and transition-metal ions has been proven to be particularly effective in tailoring the structural, optical, and electronic properties of BFO, thereby enhancing its photocatalytic activity.^16–18,20,28–30^

Special interest is put to praseodymium (Pr­(III)) and cobalt (Co­(III)) codoping, as it provides substitution at both A-site (Bi­(III)) and B-site (Fe­(III)) of the perovskite lattice. Such codoping induces lattice distortions, oxygen vacancies, and defect states that lead to band gap narrowing and suppression of electron–hole recombination.^17,18,20,28–30^ According to previous studies, rare-earth substitution stabilizes the crystal structure and tunes the optical absorption, while transition-metal substitution modifies electronic states, thus introducing intermediate energy levels that facilitate visible light absorption.^17,18,28–30^ Consequently, the synergistic effect of Pr–Co codoping is expected to significantly enhance the photocatalytic reduction of Cr­(VI).

Most reported studies focus on finding the structural and optical properties of photocatalysts^31–33^ using conventional techniques such as photoluminescence (PL), UV–vis spectrophotometry, or electrochemical analyses, without paying attention to the influence of their thermal behavior on photocatalytic performance.^34–36^ Determination of thermal properties is essential since these parameters are closely related to charge carrier transport, recombination dynamics, and long-term stability of photocatalysts.

In this work, the combined investigation of thermo-optical and transport characterization is performed to provide information about their contribution to photocatalytic reduction of pollutants. Thus, the aim of the study is to develop a combined photothermal spectrometer that integrates beam deflection spectrometry (BDS) and photopyroelectric (PPE) calorimetry for characterization of pure and Pr–Co codoped BFO photocatalysts. The material’s applicability is further tested by monitoring the process of photocatalytic Cr­(VI) reduction. The BDS and PPE integrated system overcomes the limitations of single-technique measurements and provides comprehensive and simultaneous multiparameter analysis of complex materials to obtain their complete physical characterization within a single measurement with high sensitivity and high accuracy of analysis. The application of the combined BDS and PPE technique enables analysis with reduced uncertainties in the determination of desired parameters that occur when using individual methods for studying different parts of a sample for different properties at different times.^37–40^ This study is then focused on investigation of pure and Pr–Co codoped BFO nanomaterials by the use of integrated BDS and PPE system to determine their thermo-optical and transport properties.^41,42^ The crystallographic phase, morphology, and chemical bonding were characterized using powder X-ray diffraction (PXRD), scanning electron microscopy with energy-dispersive X-ray analysis (SEM–EDX), and Fourier-transform infrared spectroscopy (FTIR). Optical absorption properties were examined by UV–vis diffuse reflectance spectroscopy. X-ray photoelectron spectroscopy (XPS) was employed to analyze the surface chemical composition and oxidation states of the elements in the synthesized materials. Finally, a correlation between heat transport and photocatalytic activity is found. The photocatalytic performance of the synthesized materials was evaluated by monitoring the reduction of Cr­(VI) to Cr­(III) under natural sunlight as a model reaction for environmental remediation. It indicates that band gap narrowing, charge carrier dynamics, and thermal properties influence the overall photocatalytic performance, and an optimal doping concentration is identified, at which enhanced visible-light absorption is achieved without excessively increased charge carrier recombination. These findings not only improve the fundamental understanding of doped BFO systems but also highlight the importance of integrating photothermal characterization with photocatalytic evaluation for the rational design of high-performance photocatalysts.

## 2 Materials and Methods

### 2.1 Sample Preparation

Pure and Co–Pr codoped bismuth ferrite nanomaterials (Bi_(1–*x*)_Pr_*x*_Fe_(1–*x*)_Co_*x*_O_3_, where *x* = 0.0, 0.02, 0.04, 0.06, 0.08, 0.10) were synthesized by the coprecipitation method. The used precursors were bismuth nitrate pentahydrate (Bi­(NO_3_)_3_·5H_2_O) (Sigma-Aldrich; >98%), iron nitrate nanohydrate (Fe­(NO_3_)_3_·9H_2_O) (Sigma-Aldrich; >98%), praseodymium­(III) nitrate hexahydrate (Pr­(NO_3_)_3_·6H_2_O) (Sigma-Aldrich; 98%), and cobalt nitrate hexahydrate (Co­(NO_3_)_2_·6H_2_O) (Sigma-Aldrich; 98%). The used chemicals were of analytical grade and therefore did not require any additional purification.

For the synthesis of Pr–Co codoped bismuth ferrite, bismuth nitrate pentahydrate was first dissolved in double-distilled water [DDW]. Independently, a dilute solution of nitric acid (HNO_3_) (Qualigens, 99%) was prepared (∼4.4 M) and added to the 2 M bismuth nitrate solution to make it soluble. At the same time, iron nitrate nonahydrate was dissolved in 40 mL of DDW, whereas cobalt nitrate hexahydrate and praseodymium nitrate hexahydrate were dissolved in 10 mL of DDW (Table 1). The four solutions were then mixed while being constantly stirred for an hour to achieve a uniform mixture. Dropwise addition of ammonia solution (Sigma-Aldrich, 28–30% NH_3_ basis) was made until the pH of the solution was more than 11 to cause precipitation of doped bismuth ferrite. The precipitate was centrifuged (Microprocessor Micro Centrifuge, Max speed: 16000 rpm, Max RCF: 16600 “G”) multiple times with DDW and ethanol to remove any unreacted impurities. The precipitate was further purified, dried in an oven (Hot air oven, MFG SM Scientific Instrument PLT) at 80 °C, and sintered (high-temperature furnace, Jupiter, India) at 600 °C for 2 h to obtain the final Pr–Co codoped BFO material.^16^ Synthesized powders were stored and packed for future characterization and analysis. Figure 1 illustrates the synthesis route schematically, while Figure 2 displays the photographs of pure and doped BFO materials. A noticeable variation in color is observed with increasing Co–Pr doping, indicating changes in optical absorption, which coincide with the results obtained by UV–vis analysis.

**Table 1:** Amount of Salts Used for Preparation of BFO Based Photocatalysts with Different Dopants Concentrations

	Bi(NO_3_)_3_·5H_2_O (g)	Fe(NO_3_)_3_·9H_2_O (g)	Co(NO_3_)_2_·6H_2_O (g)	Pr(NO_3_)_3_·6H_2_O (g)
BFO	9.30	7.75	NA	NA
BF2	9.16	7.63	0.11	0.17
BF4	9.01	7.50	0.23	0.34
BF6	8.86	7.38	0.34	0.51
BF8	8.70	7.25	0.45	0.68
BF10	8.55	7.12	0.57	0.85

**Figure 1:**
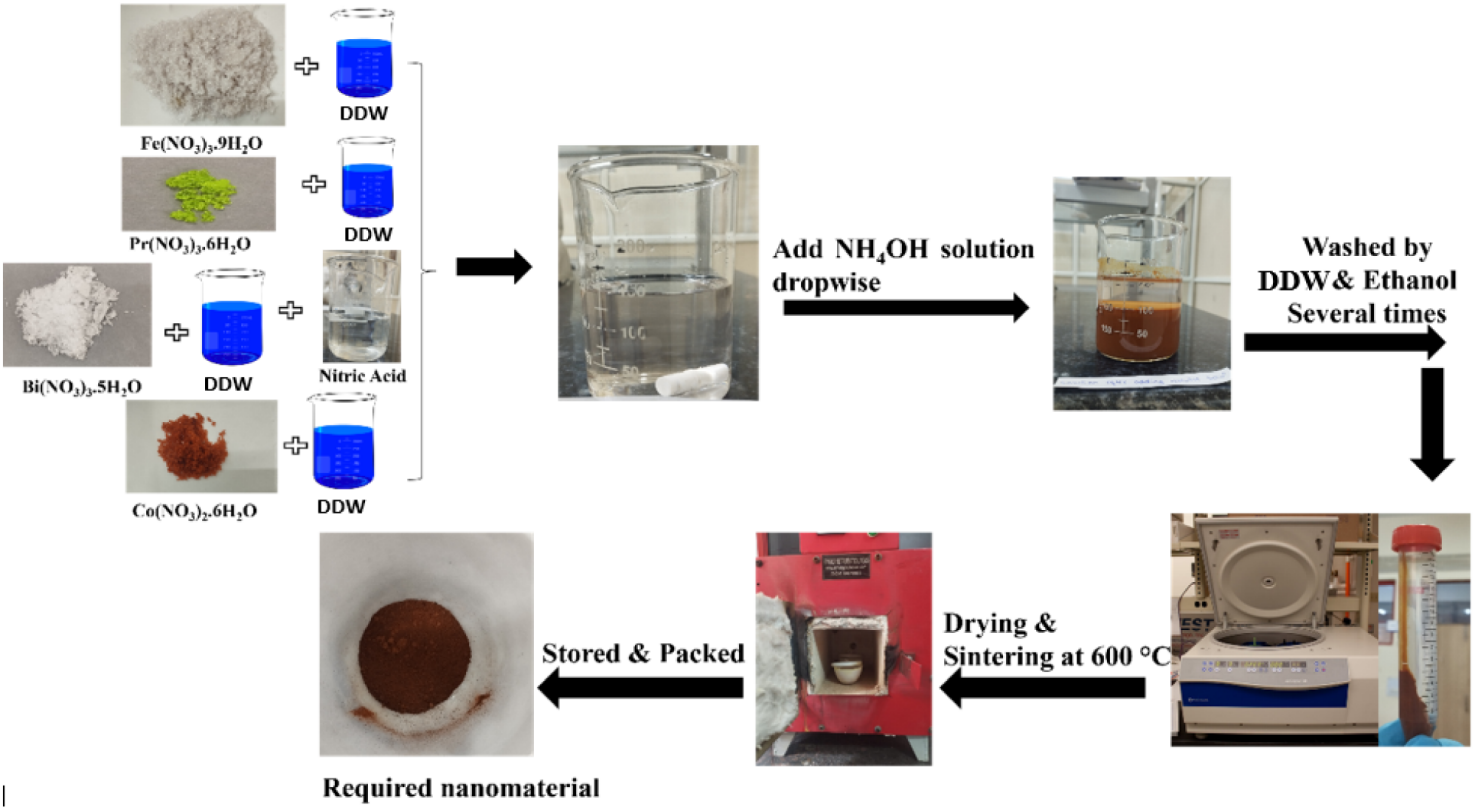
Synthesis process of pure and Co–Pr doped BFO.

**Figure 2:**
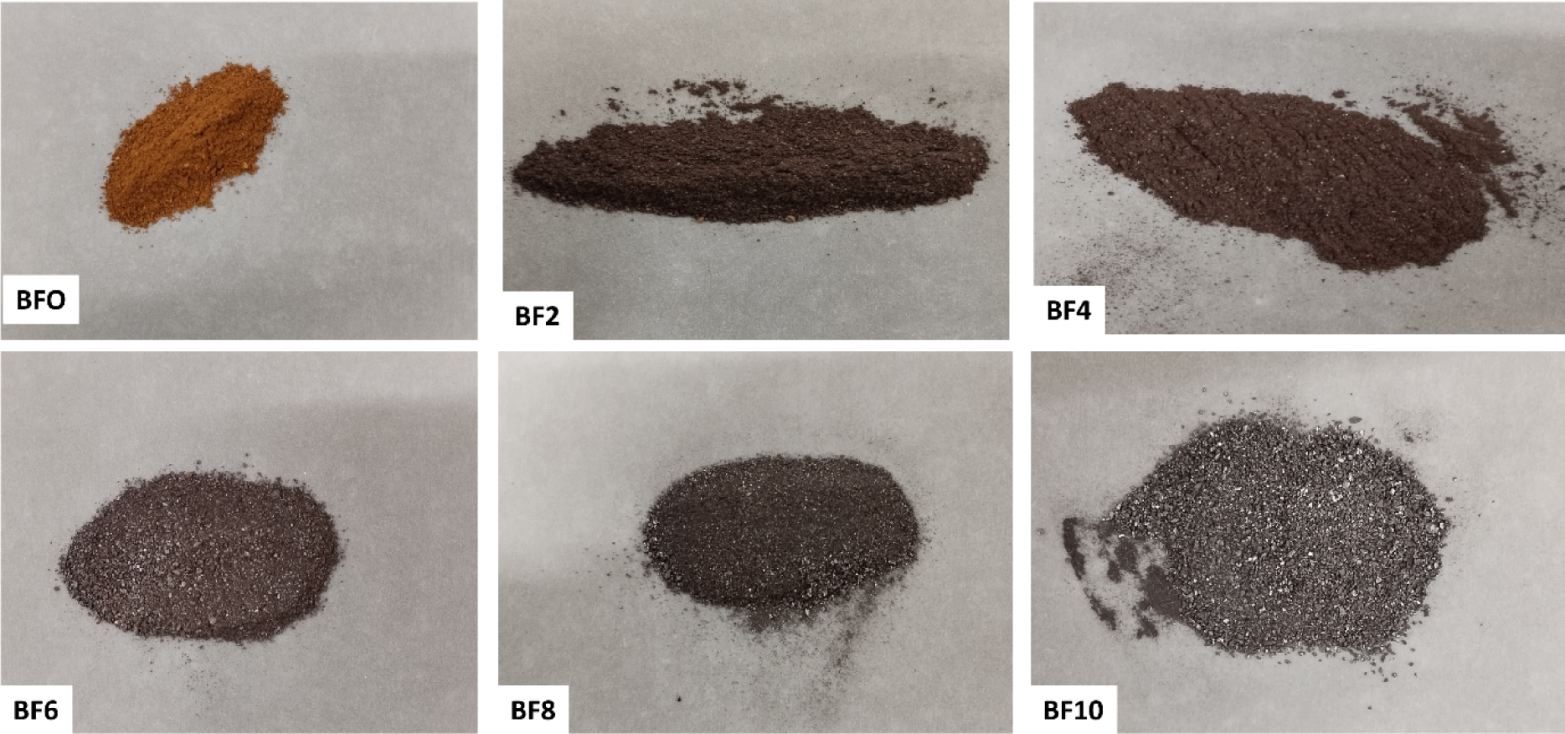
Photographs of pure and Pr–Co codoped BFO nanomaterials.

### 2.2 Photocatalytic Reduction of Cr­(VI)

The photocatalytic performance of the prepared pure and Co–Pr codoped BFO nanomaterials was determined by monitoring the reduction process of hexavalent chromium [Cr­(VI)] to trivalent chromium [Cr­(III)]. The Cr­(VI) reduction process was observed using the 1,5-diphenylcarbazide (DPC) method, which is a well-established colorimetric technique for the selective detection of Cr­(VI).^4^

The photocatalytic process was monitored under solar light irradiation (Figure 3). Pr–Co codoped BFO materials absorb photons with energy equal to or greater than their band gap, leading to excitation of electrons from the valence band (VB) to the conduction band (CB). We deal with the simultaneous generation of photogenerated electrons and holes:

1 $$\mathrm{Pr-Co-BFO}+h\nu \to e_{\mathrm{CB}}^{-}+h_{\mathrm{VB}}^{+}$$

**Figure 3:**
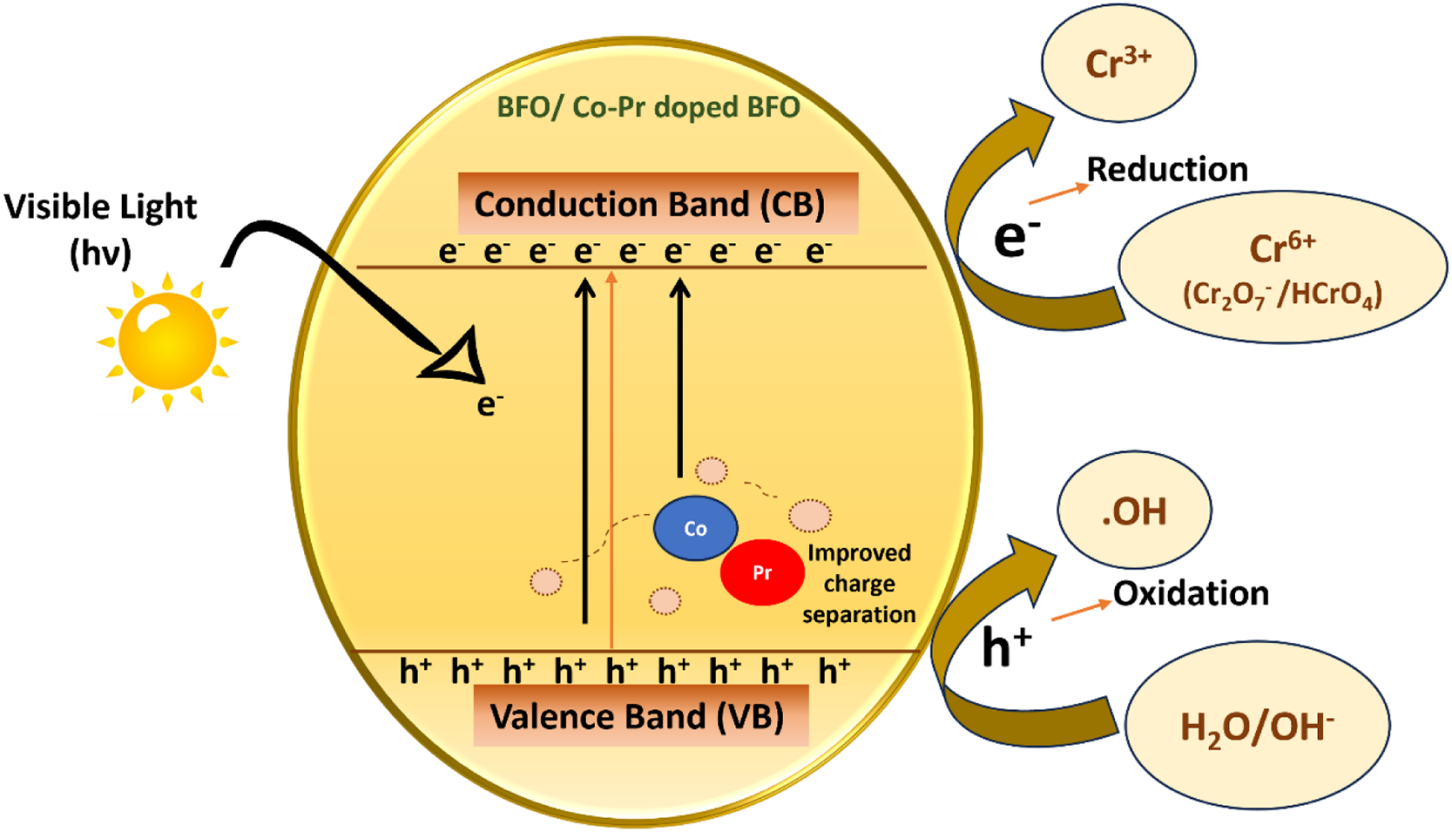
Visible-light-driven photocatalytic reduction of Cr­(VI) to Cr­(III) using pure and Co–Pr codoped BFO material.

In acidic aqueous media, hexavalent chromium predominantly exists as hydrogen chromate ions (HCrO^–^_4_), which can adsorb onto the photocatalyst’s surface through electrostatic interactions. The conduction band potential of Pr–Co codoped BFO is sufficiently negative to enable the direct reduction of adsorbed Cr­(VI) species. Consequently, photogenerated electrons accumulated in the CB participate in the multielectron reduction of Cr­(VI) to the less toxic trivalent chromium (Cr­(III)):

2 $$HCrO_{4}^{-}+7H^{+}+3e_{CB}^{-}\to Cr^{3+}+4H_{2}O$$

Simultaneously, the photogenerated holes in the valence band are consumed by oxidation reactions involving water molecules or surface-adsorbed hydroxyl groups, producing reactive intermediates and preventing rapid electron–hole recombination:

3 $$H_{2}O+h_{VB}^{+}\to •OH+H^{+}$$

Or

4 $$OH^{-}+h_{VB}^{+}\to •OH$$

The presence of surface hydroxyl groups facilitates hole consumption and enhances interfacial charge transfer processes. Pr–Co codoping modifies the electronic structure of BFO, leading to enhanced visible-light absorption and improved charge separation, as reflected by the observed changes in carrier lifetime. The reduced recombination probability increases the availability of CB electrons for Cr­(VI) reduction.^31^

#### 2.2.1 Preparation of Cr­(VI) Stock Solution

Potassium dichromate (K_2_Cr_2_O_7_, Alfa Aesar, 99% purity) was used as a source of Cr­(VI).

Concentrations of Cr­(VI) in contaminated groundwater affected by industrial waste or industrial wastewater range from 0.1 mg/L to over 300 g/L.^43–45^ Thus, the concentration of the Cr­(VI) working solution was chosen to be within this concentration range as a standard for Cr­(VI) to Cr­(III) reduction experiments.

A stock solution of 1 g/L was then prepared by dissolving 2.83 mg of K_2_Cr_2_O_7_ in 1 L of DDW. The obtained solution was further diluted to obtain a working Cr­(VI) solution of 1 mg/L.

#### 2.2.2 Preparation of Cr­(III) Stock Solution

Cr­(III) standard solution was prepared by dissolving 7.69 mg of chromium­(III) nitrate nonahydrate (Cr­(NO_3_)_3_·9H_2_O, Thermo Fisher Scientific, 99% purity) in 1 L of DDW, which results in a 1 g/L Cr­(III) stock solution. This blue-colored solution was used to verify the formation of Cr­(III).

#### 2.2.3 Preparation of 1,5-Diphenylycarbazide (DPC) Reagent

The DPC reagent was freshly prepared before use by mixing 250 mg of DPC (Sigma-Aldrich) in 50 mL of acetone. The solution was stored in a brown glass bottle to prevent degradation under light. DPC is sensitive to oxidation, and any discolored solutions were discarded. DPC was selected because it forms a stable Cr–DPC complex in an acidic medium, which enables sensitive and selective spectrophotometric detection of Cr­(VI), even at trace levels.^4^

#### 2.2.4 Formation of Cr–DPC Complex and Photocatalytic Experiments

For the photocatalytic reduction studies, 200 mL of 1 mg/L Cr­(VI) solution was mixed with 2 mL of freshly prepared DPC reagent. The solution’s pH was adjusted to a value of 1.5 using sulfuric acid. Acidic conditions are necessary both to stabilize the Cr–DPC complex and to prevent the precipitation of Cr­(OH)_3_ during the reduction process. In the present work, the DPC complexing agent was added to the Cr­(VI) solution prior to reduction studies of Cr­(VI) to Cr­(III).

On the whole process, the formed Cr–DPC complex acts as a sensitizer, enhancing light absorption and consequently facilitating charge transfer that takes part in the photocatalytic process.^46^ Furthermore, it captures the photoexcited electrons, preventing them from recombining with holes and thus improving the efficiency of the whole reduction process.^47^ Since the used photocatalysts are metal-based semiconductors, the Cr–DPC complex also serves as an accelerator of reduction kinetics of Cr­(VI) to Cr­(III).^48^ The reason for this is the easier adsorption of the Cr–DPC complex on the surface of the photocatalyst compared to Cr­(VI) ions; thus, Cr–DPC can more easily access its active sites.^49^ Finally, DPC improves the efficient transformation of Cr­(VI) to Cr­(III).^50^

The Cr–DPC complex formation involves the reduction of Cr­(VI) to Cr­(III) by DPC in an acidic environment. DPC is then oxidized to diphenylcarbazone (DPCO), which forms a purple complex with Cr­(III) that has maximum absorption at 540 nm. The reaction occurs as^51^

5 $$\begin{array}{l} \mathrm{Cr}\left(\mathrm{VI}\right)+\mathrm{DPC}\\ \mathrm{oxidation} \end{array}\to \begin{array}{l} \mathrm{Cr}\left(\mathrm{III}\right)+\mathrm{DPCO}\\ \mathrm{complexation} \end{array}\to \mathrm{purple complex}$$

A total of 200 mg of photocatalysts (pure and codoped BFO) was then added to the Cr–DPC solution, and the suspension was magnetically stirred in the dark for 1 h to establish the adsorption–desorption equilibrium.

The measurements were taken in the open, sunny space located at 45° 56′27″N, 13° 38′37″ E.

The mixture was exposed to natural sunlight (in the time period of 1 Aug’25–10 Aug’25) for 120 min under clear-sky conditions between 11:30 AM and 2:00 PM local time, with ambient temperatures ranging between 27 and 29 °C.^52^ Although direct solar irradiance measurements were not recorded during the experiments, the estimated global solar irradiance under these conditions is approximately 800–1000 W/m^2^, based on standard meteorological data for the region during the experimental period. All photocatalytic tests were conducted simultaneously or under identical sunlight conditions to ensure reliable comparative analysis. Therefore, the observed relationships between material properties and photocatalytic performance are interpreted qualitatively.

During irradiation, 3 mL aliquots were withdrawn every 10 min. The collected samples were centrifuged at 5000 rpm for 3 min. (Eppendorf Centrifuge 5804) to remove suspended catalyst particles before UV–vis analysis. The concentration of Cr­(VI) was determined by measuring the absorbance using a UV–vis spectrophotometer. The photocatalytic efficiency was calculated according to the relation:

6 $$\eta \;\left(\%\right)=\frac{\left(C_{o}-C\right)}{C_{o}}\times 100$$

where *C_o_* is the initial concentration of Cr­(VI) and *C* is the concentration at irradiation time *t*. The reduction kinetics were analyzed using the pseudo-first-order model:

7 $$\ln\left(\frac{C}{C_{o}}\right)=-kt$$

where *k* is the rate constant.

### 2.3 Characterization Methods

All of the measurements and related determination procedures were repeated three times. On this basis, the average values and their standard deviations for each determined parameter were calculated to evaluate the reproducibility of the whole analysis.

The crystalline structure of the synthesized samples was examined using the Powder X-ray Diffraction (PXRD) technique (Rigaku SmartLab, 40 kV, 40 mA, with D7teX Ultra detector) with Cu Kα radiation (λ = 1.5406 Å). Diffraction patterns were recorded in the 2θ range of 20°–70° at a scan rate of 2° min^–1^.

The vibrational modes of the samples were analyzed by Fourier Transform Infrared Spectroscopy (FTIR) (PerkinElmer Spectrum 100 with PIKE GladiATR), where spectra were obtained with at least 10 scans in the range of 450–3200 cm^–1^.

The optical absorption properties, including monitoring the reduction process of Cr­(VI), were investigated using a UV–vis diffuse reflectance spectrophotometer (UV–vis DRS) (PerkinElmer Lambda 650, USA) in the wavelength range of 250–500 nm.

The surface morphology and chemical composition were characterized by scanning electron microscopy coupled with energy-dispersive X-ray analysis (SEM-EDX) (JEOL JSM-7100F, field emission gun), operated at an accelerating voltage of 15 kV. Prior to SEM observation, powder samples were ultrasonically dispersed in ethanol and deposited on conductive substrates. Despite dispersion, some agglomeration was observed, which is common for ferrite-based oxide nanoparticles synthesized by chemical methods.

XPS measurements were performed using a monochromated Al Kα X-ray source (Thermo Scientific) and an SES R3000 electron energy analyzer (Scienta). Prior to measurement, powder samples were pressed into pellets (diameter: 6 mm) using a hydraulic press under an applied load of 3 tons for 1 min. All measurements were carried out at room temperature.

The thermo-optical and transport parameters (thermal diffusivity and conductivity, energy band gap, and carrier lifetime) were determined using a home-built photothermal spectrometer that integrates BDS with the PPE technique. In such a system, a near-ultraviolet (UV) solid-state laser (Oxxius, Model LBX-375–200-HPE-PPA, France) with an output power of 100 mW and an output wavelength of 377 nm was chosen as an excitation beam (EB) source. Its emission line coincides with the samples’ absorption spectra to ensure high sensitivity for both BDS and PPE measurements.

EB was intensity modulated within the frequency range of 3–200 Hz using a lock-in amplifier (Stanford Research Systems, Model SR830 DSP) and directed onto a 50:50 plate beam splitter (Thorlabs, UVFS, AR coating: 350–1100 nm), where it was split into a transmitted forward beam and a reflected downward beam. The first of these was further directed to the PPE module, whereas the second one was directed to the BDS part of the whole system (Figure 4a–b). In the BDS module, the EB was defocused by a biconvex lens with a focal length of 100 mm (Lens 3, AR coated: 350–700 nm; Edmund Optics) to form a 2 mm diameter spot on the sample surface that was positioned onto a motorized 3D translation stage (CVI, Model2480M/2488) to allow precise alignment of the system to optimize the BDS signal. A continuous He–Ne laser with a 543.1 nm output wavelength and 2 mW output power (Melles Griot, Model 25-LGR-393–230) was used as a probe beam (PB) source. The PB was focused by a biconvex lens (Lens 2, AR coated: 350–700 nm; Edmund Optics) to have a radius at its waist of 40 mm over the sample’s surface to ensure a 1D configuration of the setup.^53^ The PB was aligned to skim the sample’s surface. The change in PB intensity caused by its interaction with temperature oscillations induced by the EB over the sample’s surface was recorded by a position-sensitive detector (RBM-R, Braumann GmbH, Model C30846E), equipped with an interference filter (center wavelength: 632 nm; Edmund Optics) that converted the optical signal into the electrical one. The signal was collected and processed through a lock-in amplifier (Stanford Research Systems, Model SR830 DSP).

**Figure 4:**
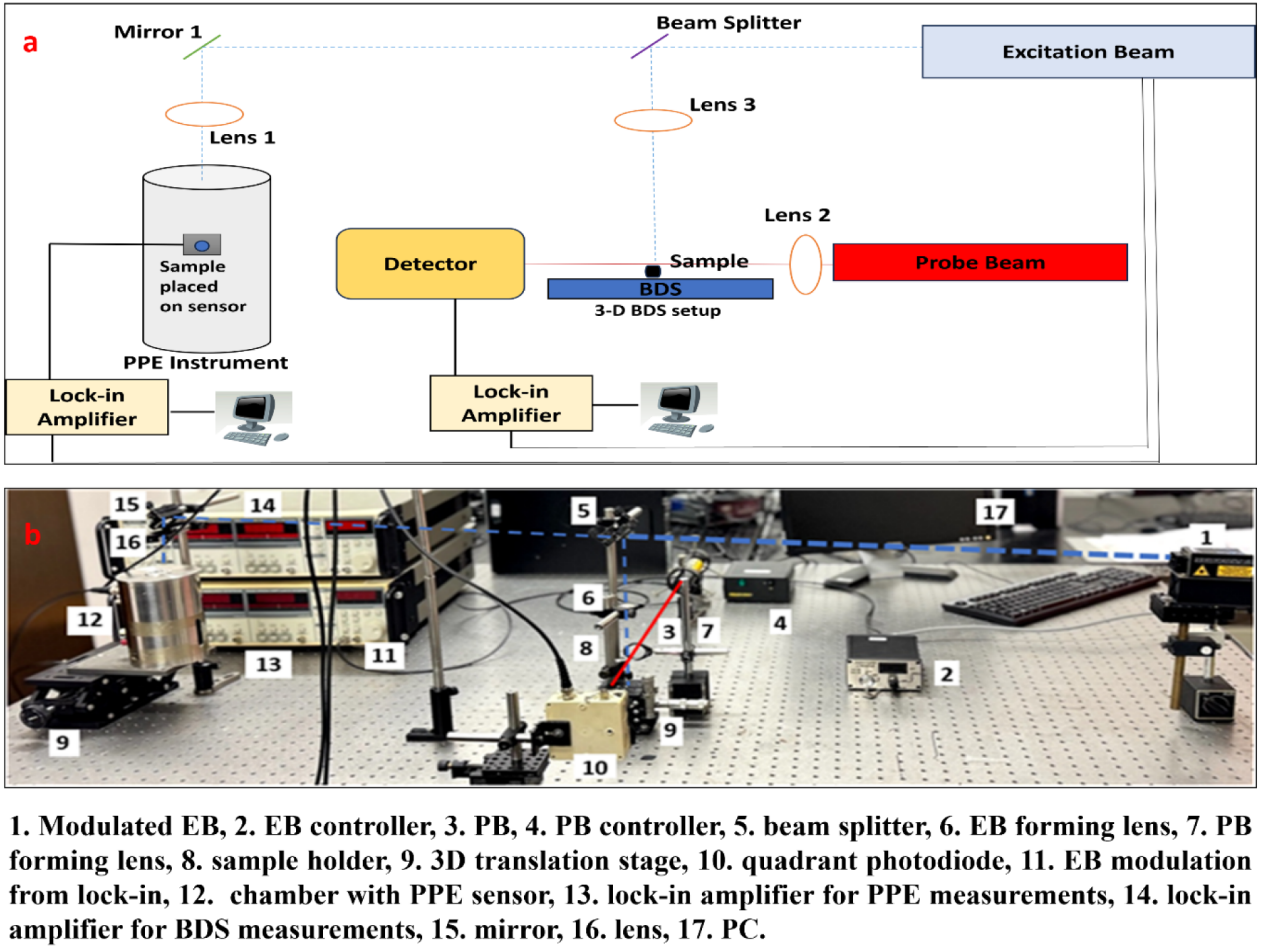
Scheme (a) and photograph (b) of the combined BDS and PPE experimental setup.

In the PPE module, the transmitted part of EB radiation is reflected by a broadband dielectric mirror (Thorlabs BB1-E02, 350–1100 nm) to be formed by a biconvex focusing lens (Lens 1, AR coated: 350–700 nm; Edmund Optics) and further directed perpendicularly onto the surface of the sample through a 5 mm thick quartz window (QW) placed over a pyroelectric sensor. A small layer of viscous high-vacuum silicon grease was applied onto it to make good thermal contact between the sensor and the sample. The sensor consists of a LiTaO_3_ single crystal with Cr–Au electrodes deposited on both its sides. It is 400 μm thick and has a surface area of 15 × 15 mm^2^. The QW is transparent to the visible and near-infrared spectrum and is positioned in front of the sensor to minimize thermal artifacts resulting from infrared radiation, thus ensuring thermal isolation. The thermal oscillations generated in the sample by a modulated EB induce voltage changes across the pyroelectric sensor that are further measured by a lock-in amplifier (Stanford Research Systems, Model SR830 DSP).

In the case of both BDS and PPE measurements, the lock-in amplifiers are connected to a PC for collection of the amplitude and phase of either the BDS or PPE signal as a function of EB modulation frequency by MATLAB software.

## 3 Results and Discussion

### 3.1 Crystalline Structure Analysis

PXRD patterns for pure BFO and modified samples with compositions Bi_(1–*x*)_Pr_*x*_Fe_(1–*x*)_Co_*x*_O_3_ (for *x* = 0.02, 0.04, 0.06, 0.08, and 0.10) are shown in Figure 5. All the samples exhibit clear diffraction peaks, indicating good crystallinity throughout the entire doping series. The diffraction peaks have been indexed to the rhombohedral distorted perovskite structure with space group *R*3*c* as per JCPDS Card No. 71-2494.^17^ Prominent peaks are located at 2θ values indexed for planes (012), (104), (110), (006), (202), (024), (116), (122), (214), (300), (208), and (220). The preservation of these diffraction planes in all the samples indicates that the perovskite structure is not compromised by Pr­(III) and Co­(III) doping, and no signs of impurity phases such as Bi_2_O_3_ or Fe_2_O_3_ were observed, indicating high phase purity.

**Figure 5:**
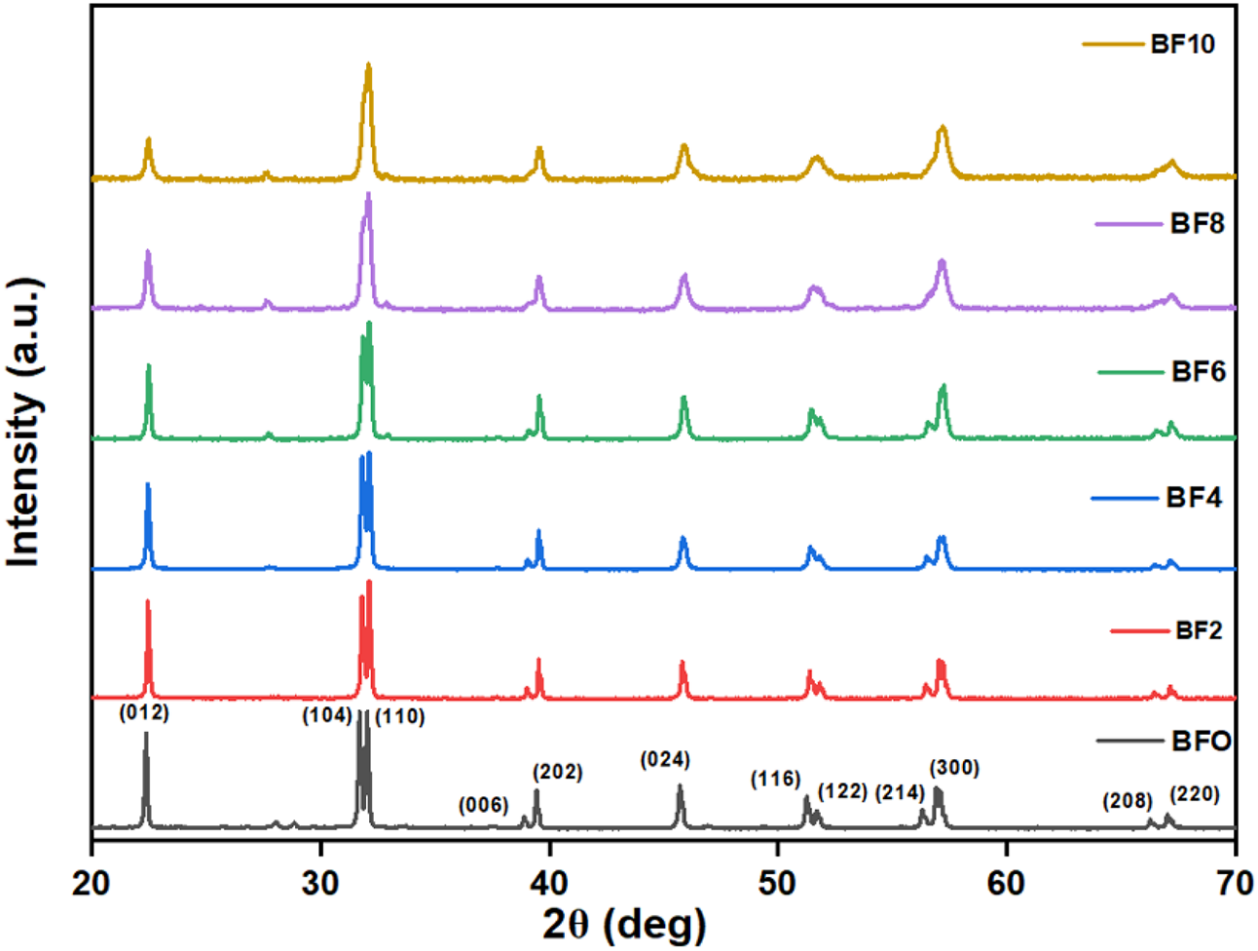
PXRD graphs of BFO and Co–Pr doped BFO Bi_(1–*x*)_Pr*_x_* Fe_(1–*x*)_Co*_x_* O_3_ (for *x* = 0.02, 0.04, 0.06, 0.08, and 0.10).

Furthermore, a low-intensity diffraction peak is observed around 2θ ∼ 28° in all samples, including undoped BFO, indicating that it is not related to dopant-induced contamination. This peak can be attributed to a low-intensity reflection associated with the rhombohedrally distorted BFO perovskite structure, which has been reported previously in BFO systems.^54^ Also, the intensity of this peak does not increase with dopant concentration. These observations confirm that the peak does not originate from secondary phases or contamination.

A slight shift in peak positions with increasing concentration of doping is observed. This transformation is attributed to the substitutional replacement of Pr­(III) (ionic size ∼1.13 Å) for Bi­(III) (ionic size ∼1.17 Å) in the A-site and Co­(III) (ionic size ∼0.745 Å) for Fe­(III) (ionic size ∼0.645 Å) in the B-site of the perovskite lattice. Substitutional ions induce internal strain in the lattice and therefore result in peak shift and broadening. These structural distortions would also mean successful doping at the atomic level, which would change the electronic structure and influence thermal transport as well as photocatalytic functionality of the material.^17^

Some peak broadening can also be observed in the PXRD patterns with increased doping. To perform the quantitative analysis, the surface areas of samples’ diffraction peaks were fitted using a Gaussian function, and subsequently, the full width at half-maximum (fwhm) was calculated. For example, the peak indexed to the (012) plane clearly demonstrates an increasing fwhm as the doping increases. In samples BF2, BF4, BF6, BF8, and BF10, the fwhm equals 0.135, 0.168, 0.175, 0.252, and 0.288, respectively. This quantitatively describes the fact that peak broadening follows the trend of increased doping. Furthermore, there are some peaks that appear as two separate peaks in the pristine BF0 sample and merge into a single peak at higher doping amounts: such are the peaks assigned to the (104) and (110) planes in BF0, which merge into a single peak in BF8 and BF10. Bismuth ferrite normally crystallizes in a low-symmetry space group (*R*3*c*). Certain reflections, which would be equivalent in higher-symmetry space groups, are here nondegenerate; hence, peak splitting occurs. When dopants are introduced, this brings in peak broadening, and when peak separation becomes smaller than the fwhm, the reflections appear merged.

In order to further investigate the influence of doping on crystallite size, the Debye–Scherrer equation is applied to the most intense diffraction peak:

8 $$D=\frac{k\lambda }{\beta \cos\theta }$$

where *D* is the average crystallite size, *K* is the shape factor (0.9), λ is the wavelength of the X-ray (1.5406 Å for Cu Kα), β is the full width at half-maximum (fwhm) of the peak in radians, and θ is the Bragg angle. The calculated average crystallite sizes are presented in Table 2. The initial increase in crystallite size was observed at 2% doping, which may be attributed to increased grain growth based on minor strain relief within the grains and greater mobility of the species during the sintering process.^17^ With an increase in dopant concentrations, an increasing crystallite size reduction is seen, with the smallest crystallite size found for the highest doping concentration of *x* = 0.10. These reductions may be due to increased lattice strain and defect formation resulting from higher amounts of dopant incorporation, which likely hinders crystalline growth during thermal treatment.^55^ Such lattice defects have the capability to introduce local distortions and disrupt long-range order, inhibiting the clustering of crystallites. From a photocatalytic perspective, this reduction in crystallite size with an increase in dopant loading may be advantageous because it can facilitate improved light absorption and higher interaction with the reactants during the reduction of Cr­(VI).^56^ So, the PXRD results confirm that Pr and Co are successfully doped into the lattice of BFO without disrupting the parent perovskite structure. The resulting crystallite size evolution and peak shifts are a confirmation that doping induces structural changes, which are expected to influence the optical, thermal, and photocatalytic properties of the materials.

**Table 2:** Average Crystallite Size and Optical Band Gap of Pure BFO and Co–Pr Codoped BFO Samples

Samples	Atomic percent of doping (*x*) (wt %)	Average crystallite size (nm)	Band gap (eV)
BFO	0.00	58.17	2.05 ± 0.05
BF2	0.02	66.57	1.75 ± 0.05
BF4	0.04	45.26	1.70 ± 0.05
BF6	0.06	42.06	1.45 ± 0.04
BF8	0.08	26.74	1.40 ± 0.04
BF10	0.10	24.42	1.27 ± 0.04

### 3.2 Optical Properties Determination

The optical absorption behavior and band gap values of pure and codoped BFO were evaluated by the use of UV–vis DRS.

The values of direct and indirect band gaps of the semiconductor can be found on the basis of photocatalysts’ diffuse reflectance spectra using Kubelka–Munk theory^57^ according to which the relationship between the band gap and the absorption coefficient can be obtained by the use of equation:

9 $${\left(\alpha h\nu \right)}^{n}\propto h\nu -E_{g}$$

where *E*_*g*_ is the band gap of allowed transitions (eV), *h* is Planck’s constant (6.63 × 10^–34^ J s), ν is the frequency of light (s^–1^), *n* is the number characterizing the transition process (*n* = 0.5 and *n* = 2 are related to indirectly and directly allowed transitions, respectively). The band gaps of BFO-based materials were then found by extrapolating linear parts of graphs described by eq 9 to intercept the horizontal axis ((α*h*ν)^η^ = 0).

BFO is a photocatalytic material with an indirect band gap, whose value is in the range of 2.0–2.8 eV, depending on the preparation method and the form of the material (nanoparticles or thin film).^58,59^

The absorption coefficient was obtained from the DRS data (Figure 6a) while Figure 6b shows the corresponding Tauc plots. All samples absorb in the 250–800 nm wavelength range, with a clear red shift of the absorption edge as the dopant’s concentration increases. The red shift is an indicator of enhancement in the ability of material to absorb visible light, which is desirable for photocatalytic applications.^20,28,29^ The shift is greater in the doped samples compared to the undoped BFO, suggesting that Pr and Co codoping strongly alters the electronic structure. The calculated band gap values are shown in Table 2. The results demonstrate a gradual reduction in band gap with increasing Pr and Co content. The introduction of intermediate energy levels within the band structure may result from dopant-induced lattice distortion, oxygen vacancies, and changes in the Fe–O–Fe exchange interactions.^28,29^ The lowest band gap of 1.27 eV obtained for the 10% doped sample is an indication of a broad extension of optical absorption in the visible region, which should increase the photocatalytic activity under visible or solar light illumination. In addition, a lower band gap will facilitate easier excitations of electrons from the valence to the conduction band, which would improve charge carrier generation and mobility.^20,60^ However, excessive narrowing of the band gap (e.g., as in BF10) will also form recombination centers or localized states that can be traps for charge carriers, decreasing their lifetimes and thus, affecting photocatalytic performance.^60^ Therefore, a balance between enhanced visible light absorption and lower recombination needs to be found. A moderate doping levels (e.g., 6–8%) may be an effective compromise, which is in good agreement with results obtained from structural and FTIR analyses (see Section 3.1 and Section 3.3).

**Figure 6:**
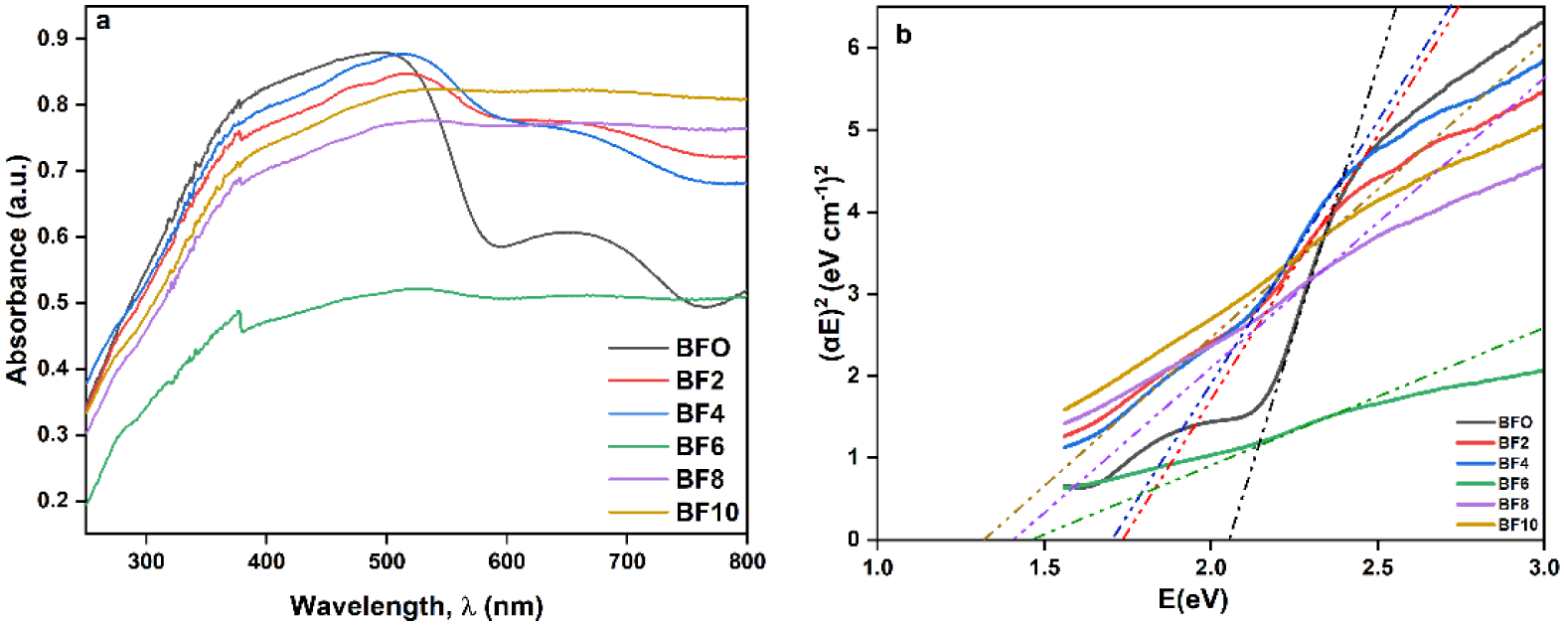
Absorbance spectra (a) and Tauc plots (b) of BFO and Co–Pr doped BFO Bi_(1–*x*)_Pr*_x_* Fe_(1–*x*)_Co*_x_* O_3_ (for *x* = 0.02, 0.04, 0.06, 0.08, and 0.10).

The obtained values of the band gap for pure and Co–Pr codoped BFO coincide with literature data,^28–62^ where the band gap of pure BFO was found to be between 2.10 and 2.70 eV. By only Pr doping, the band gap can be reduced to the level of 2.27–2.44 eV, whereas only Co doping leads to a decrease in the band gap value to 1.59 eV. This value can be further reduced by performing codoping. For example, in systems similar to Co–Pr codoped BFO such as Co–Sm codoped BFO, the band gap is significantly reduced down to the value of 1.40 eV. However, the band gap values strongly depend on dopant concentration, material form, as well as the preparation procedure.

### 3.3 Organic Functional Group Identification

FTIR spectroscopy was employed to identify functional groups and confirm the presence of characteristic metal–oxygen vibrational modes in undoped and codoped BFO samples. FTIR transmittance spectra of pure BFO and Pr–Co codoped BFO samples (BF2, BF4, BF6, BF8, BF10), recorded in the range 400–4000 cm^–1^, are presented in Figure 7. All the spectra show strong absorption bands in the low wavenumber region of 550–600 cm^–1^, which are due to the Fe–O stretching vibrations of FeO_6_ octahedra. This is a confirmation of the successful formation of the perovskite BFO structure, where the B-site of the ABO_3_ lattice is filled by Fe.^14^ The presence of this band in all the codoped samples shows that octahedral coordination of Fe is maintained even after the material is codoped with Co and Pr. The extensive absorption bands in the region of 1380–1650 cm^–1^ are C=O stretching vibrations and may result from adsorbed atmospheric CO_2_. These bands are more intense for samples with lower dopant concentration and decrease marginally in intensity with increase in amounts of introduced substitutes, what indicates a reduction of organic residue as doping is observed to alter the surface chemistry and maybe improve crystallinity and calcinations behavior.^18,30^ The spectra also show weak bands at 2850–2950 cm^–1^, attributed to C–H stretching vibrations. The range of 3200–3600 cm^–1^ is attributed to O–H stretching vibrations of surface-adsorbed water molecules. The gradual suppression of these bands in the highly doped samples reflects reduced moisture adsorption and improved thermal decomposition of precursor materials. A subtle change in the intensity and sharpness of the Fe–O peak with increasing doping concentration (from BF2 to BF10) is also seen. These changes can be an indicator of lattice distortion or alteration of the local bonding environment caused by substitution of Bi­(III) with Pr­(III) and Fe­(III) with Co­(III). Since Co­(III) and Pr­(III) possess slightly different ionic radii than Fe­(III) and Bi­(III), respectively, their substitution can alter bond lengths and strengths of FeO_6_ octahedra and is expressed in the slight shifting or broadening of the Fe–O vibration band.^14^ This gradual evolution of the spectral features at higher doping is consistent with the findings of the PXRD investigation and is expected to influence thermal transport behavior as well as photocatalytic performance in further studies.

**Figure 7:**
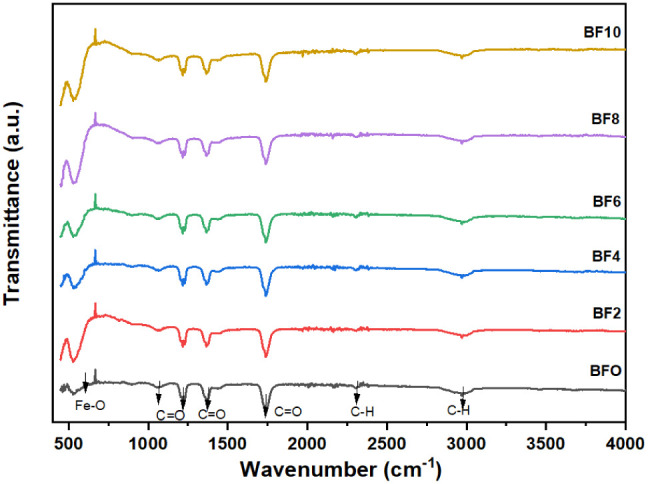
FTIR spectra of BFO and Co–Pr doped BFO Bi_(1–*x*)_Pr*_x_* Fe_(1–*x*)_Co*_x_* O_3_ (for *x* = 0.02, 0.04, 0.06, 0.08, and 0.10).

### 3.4 Surface Morphology and Chemical Composition Characterization

SEM measurements revealed severe morphological changes along the doping series. Pure BFO (Figure 8a) exhibited dense agglomeration of irregular nanostructures, indicating a strong tendency for particle clustering. After 2–4% Pr–Co codoping (Figure 8b–c), reduced agglomeration and sharper grain boundaries were observed, indicating the initial dopant effect on grain development. With 6% and 8% doping (Figure 8d–e), the morphology changed into well-developed flake-like or platelet shapes with reduced clustering, suggesting enhanced crystallinity and better grain boundary stability.^17,18,60^ However, for 10% doping (Figure 8f), reagglomeration and irregular particle sizes appeared partially, which can be ascribed to the process of dopant saturation.^28^

**Figure 8:**
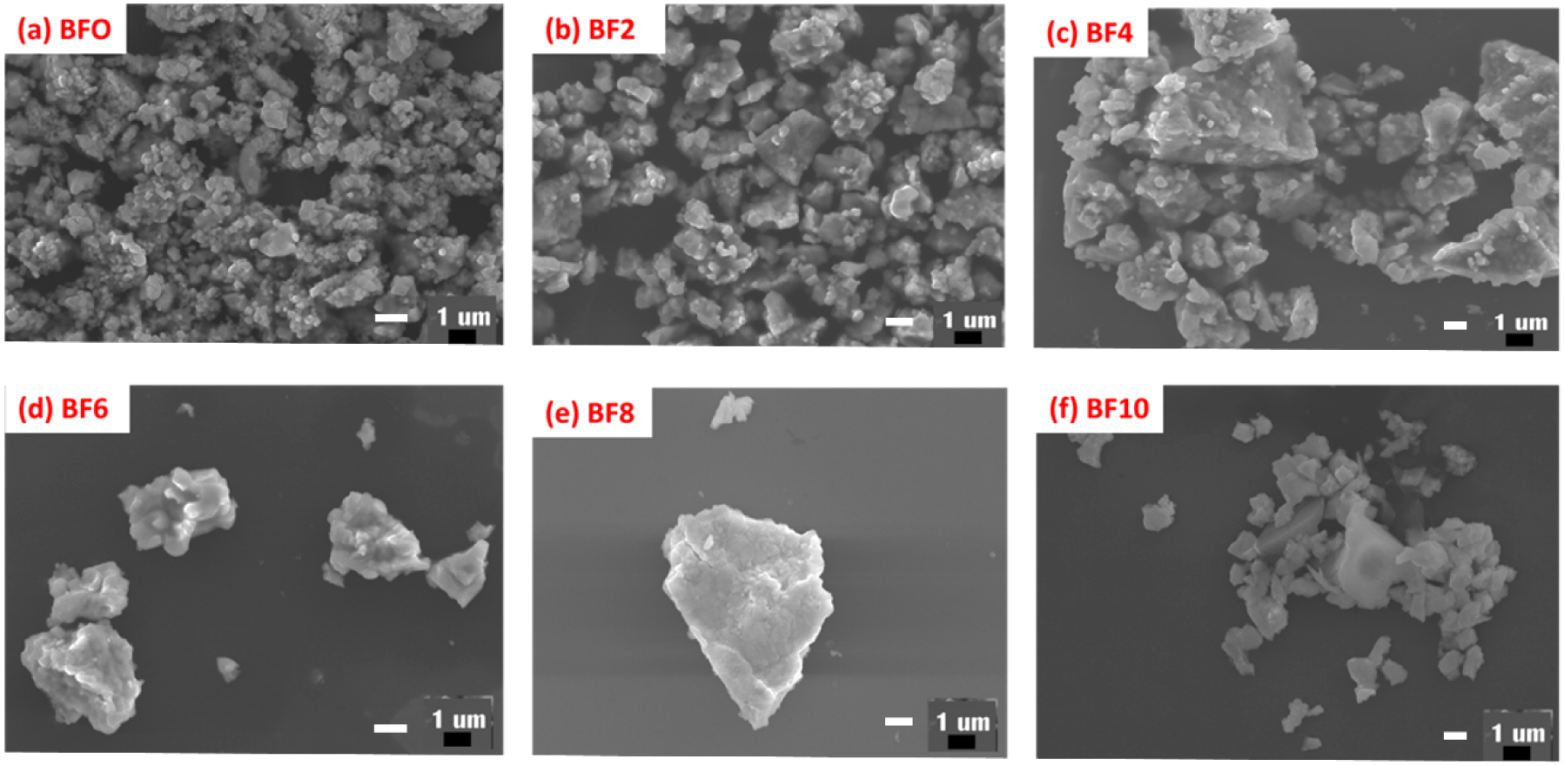
SEM images of the BFO and Co–Pr doped BFO Bi_(1–*x*)_Pr*_x_* Fe_(1–*x*)_Co*_x_* O_3_ (for *x* = 0.02, 0.04, 0.06, 0.08, and 0.10).

EDX analysis also confirmed the elemental purity of all of the samples (Figure 9a–f). BFO showed only Bi, Fe, and O peaks, suggesting phase purity. Co and Pr peaks were observed to increase in intensity with doping up to 10%, providing evidence of successful dopant incorporation. The elemental distribution was uniform, especially in BF6 and BF8. The insignificant Si signal in BF2 and BF4 is attributed to the substrate. Table 3 presents the quantified atomic percentages for BFO and BF10 samples. The detected elemental ratios of Bi, Fe, Pr, and Co are consistent with the nominal compositions within experimental uncertainty, and the uniform elemental distribution confirms successful dopant incorporation rather than surface segregation.

**Figure 9:**
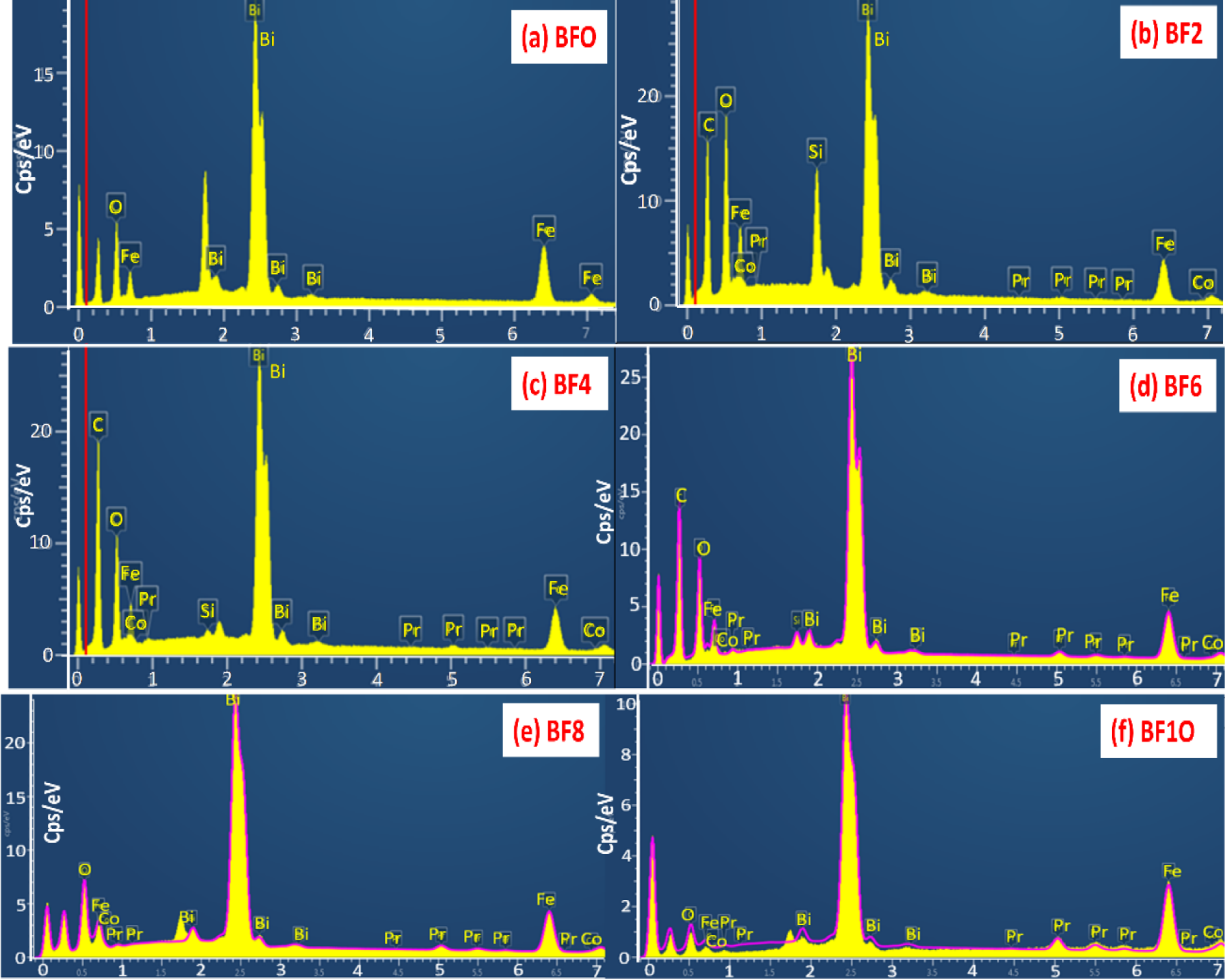
EDX analysis of the BFO and Co–Pr doped BFO Bi_(1–*x*)_Pr*_x_* Fe_(1–*x*)_Co*_x_* O_3_ (for *x* = 0.02, 0.04, 0.06, 0.08, and 0.10).

**Table 3:** Elemental Analysis of Pure and Co–Pr Codoped BFO Performed by EDX

Element	BFO (wt %)	BF10 (wt %)
Bi	53.2 ± 0.6	48.8 ± 0.4
Fe	31.3 ± 0.7	26.6 ± 0.6
O	15.5 ± 0.3	14.7 ± 0.2
Pr	-	5.4 ± 0.2
Co		4.6 ± 0.1

### 3.5 Surface Chemical Composition and Electronic States

The surface chemical composition and oxidation states of pure BFO and Pr–Co codoped BFO (BF10) were analyzed using XPS. The survey spectra of both samples confirm the presence of Bi, Fe, and O as the principal elements (Figure 10a), consistent with the expected elemental composition of a Bi-based perovskite oxide.^63^ No additional impurity-related peaks were observed within the detection limits of the XPS. The undoped BFO exhibited significant charging during measurement, resulting in spectral broadening and binding energy shifts, which limited reliable high-resolution analysis. Such behavior is commonly observed for insulating oxide materials and is consistent with previous reports for BFO.^64^

**Figure 10:**
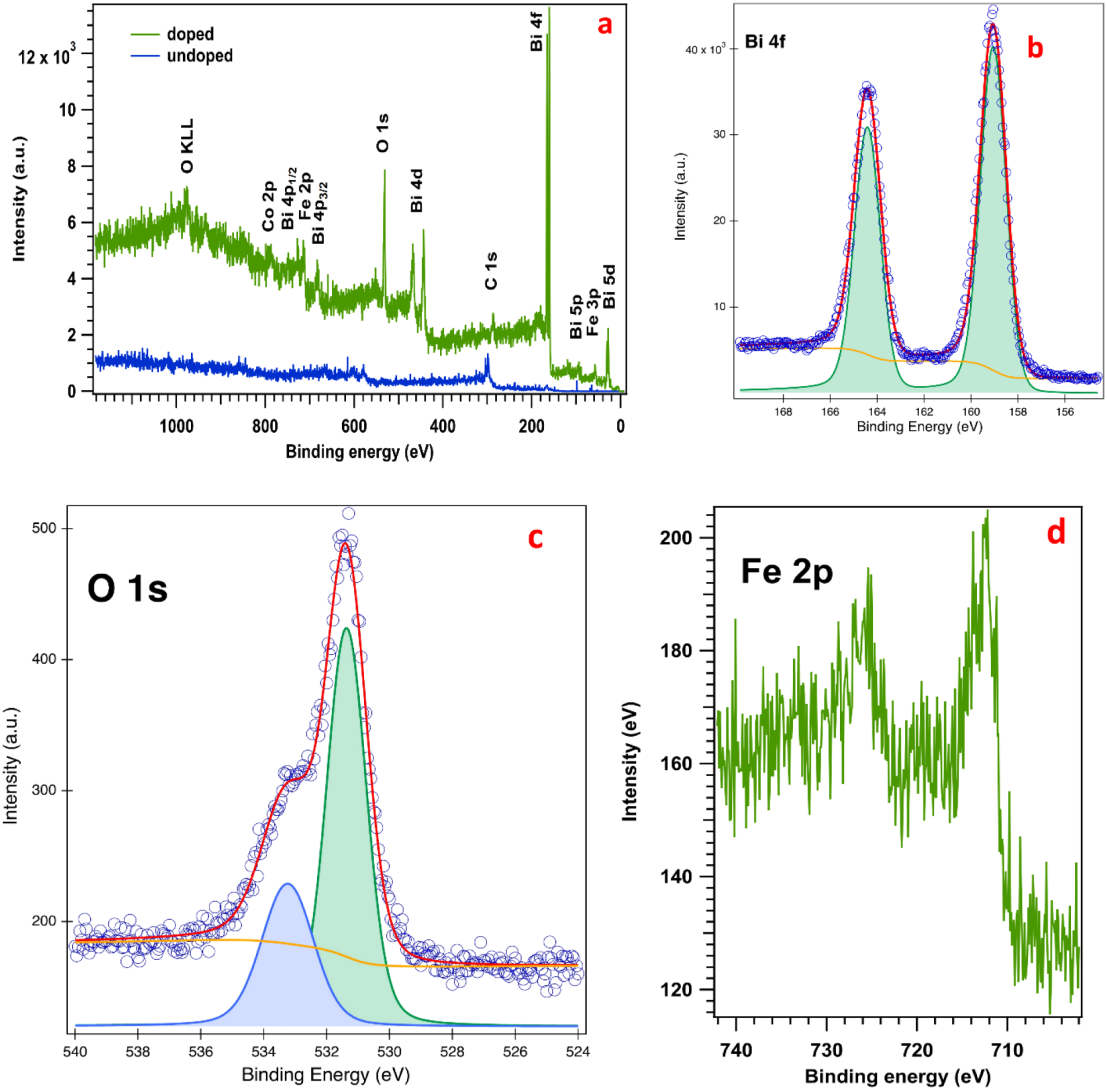
XPS survey of undoped BFO and Pr/Co-doped BFO (BF10) (a), Bi 4f core level of BF10 (b), O 1s core level of BF10 (c), and Fe 2p core level of BF10 (d).

The high-resolution Bi 4f spectrum of BF10 (Figure 10b) exhibits two well-resolved spin–orbit doublet components, Bi 4f_7/2_ and Bi 4f_5/2_, located at approximately 159.1 and 164.4 eV, respectively. The observed spin–orbit splitting of ∼5.3 eV is in excellent agreement with the reference value for bismuth and with the values reported for Bi^3+^ in BFO-based perovskites.^64,65^ The symmetric peak profiles and the absence of any feature at ∼157.0 eV confirm that no metallic Bi^0^ is present at the surface.^65^ The experimental data are well described by a two-peak Voigt fit with a linear background (Figure 10b), confirming a single bismuth chemical environment, indicating that the Bi^3+^ oxidation state is fully preserved upon Pr/Co codoping, consistent with the stabilization of the perovskite structure reported in structurally analogous Co-doped BFO systems.^66^

The O 1s core-level spectrum of BF10 (Figure 10c) was fitted with two components following a linear background subtraction. The dominant component (peak 1) is centered at approximately 531.36 eV and is attributed to lattice oxygen in the perovskite framework, associated with Bi–O and Fe–O bonds.^67,68^ These results are consistent with lattice oxygen positions reported for BFO and related perovskite oxides, where the metal–oxygen bond contribution typically falls in the range of 529.5–531 eV.^69^ A secondary, broader component (peak 2) at approximately 533.23 eV is assigned to surface hydroxyl groups and/or adsorbed oxygen species inevitably present on oxide surfaces exposed to ambient conditions prior to loading.^70,71^ It should be noted that, as demonstrated by Idriss,^71^ XPS O 1s signals in the 531–532 eV range on ex situ powder samples are most appropriately attributed to surface hydroxyls rather than to oxygen vacancies, since photoelectron spectroscopy cannot generate a signal from a missing atom and oxide surfaces oxidize rapidly upon air exposure. The relative intensity of the lattice oxygen component confirms the bulk perovskite character of BF10, and the two-component fit is sufficient to describe the experimental data without invoking additional oxygen vacancy contributions.

The Fe 2p high-resolution spectrum of BF10 (Figure 10d) shows two broad features centered near ∼711 eV and ∼724 eV, corresponding to the Fe 2p_3/2_ and Fe 2p_1/2_ spin–orbit components, respectively, with a spin–orbit splitting of approximately 13 eV. The position of the Fe 2p_3/2_ component at ∼711 eV is consistent with Fe^3+^ in an oxide environment,^72,73^ in agreement with the known Fe^3+^ valence state in single-crystal BFO, as established by Kozakov et al.^74^ through high-resolution Fe 2p, 3s, and 3P XPS. However, due to the inherently complex multiplet structure of Fe^3+^, arising from the 2p^5^3d^*n*^ final state configuration, and the relatively low signal-to-noise ratio of the present spectrum, a reliable quantitative peak deconvolution could not be performed.^74,75^

Despite the nominal 10% Pr substitution, no Pr-related photoemission features were detected in the survey spectrum of BF10. The primary Pr photoemission line accessible under Al Kα excitation is the Pr 3d core level; however, this region partially overlaps with the Bi 4s emission at approximately 940 eV, significantly complicating any identification in a Bi-rich matrix. Furthermore, the Pr 3d oxide spectra are characterized by complex multiplet and satellite structures extending up to ∼30 eV above the main peak, which substantially reduces the effective signal-to-noise ratio and the reliable detection sensitivity at low dopant concentrations. The inherently low photoionization cross-section of the relevant Pr core levels under Al Kα radiation, combined with the dominance of the Bi-related spectral background, is consistent with Pr remaining below the practical detection threshold of laboratory XPS under the present measurement conditions. Importantly, nondetection of rare-earth dopants by XPS survey at these concentration levels has been reported in analogous doped perovskite systems and does not preclude successful incorporation of Pr into the lattice, as confirmed by complementary structural characterization.^76–78^

Similarly, no reliable high-resolution Co 2p spectrum could be obtained for BF10, as the cobalt signal was heavily obscured by noise at the nominal 10% doping level. This is consistent with the known analytical challenges associated with Co 2p XPS: the 2p spectra of late 3d transition metal cations, including Co, exhibit intrinsically complex line shapes arising from charge transfer between the metal cation and oxygen ligands, multiple splitting, and intense shake-up satellite features, all of which reduce the effective peak-to-background ratio and render meaningful peak fitting impractical in the absence of a high signal-to-noise spectrum.^75,79^

### 3.6 Thermo-Optical and Transport Properties Determination

Figure 11 presents the dependence of the phase of the PPE signal on the square root of the EB modulation frequency for pure and Pr–Co codoped BFO. The thermal diffusivities of examined samples were determined using the slopes of such dependences using Figure 11 and eq A1 (see Section A1.1). The thermal diffusivity of the pure BFO sample was calculated to be 2.19 ± 0.08 mm^2^/s, which slightly increased to the value of 2.42 ± 0.10 mm^2^/s upon 2% doping. This shows the initial enhancement in thermal transport, which may be attributed to improved crystallinity and reduced lattice defects at low dopant concentrations. However, with further increase in doping, a continuous decrease in the values of thermal diffusivity is observed as 2.14 ± 0.07 mm^2^/s at 4%, 2.10 ± 0.07 mm^2^/s at 6%, 1.91 ± 0.09 mm^2^/s at 8%, and 1.84 ± 0.06 mm^2^/s at 10%. These values suggest that excess doping somehow introduces the disorder in the structure or phonon scattering centers, which deteriorates the flow of heat since the addition of impurities creates additional interfaces that in turn introduce internal thermal resistance, making it more difficult for heat-carrying phonons to propagate through the material.^80^ This trend indicates that low-level Pr–Co doping (about 2%) improves thermal transport, perhaps by reducing grain boundary resistance, which may be associated with improved structural ordering at low dopant levels. Changes in the electronic structure may also influence energy transport processes; however, thermal diffusivity is primarily governed by phonon transport within the lattice. Therefore, the observed variations are mainly attributed to structural disorder and scattering effects.^81^ It should also be highlighted that there is no correlation between the thermal diffusivity trend and the photocatalytic activity of the materials. Thus, thermal transport does not seem to play an independent role but needs to be considered together with other parameters such as optical absorption and charge carrier dynamics.

**Figure 11:**
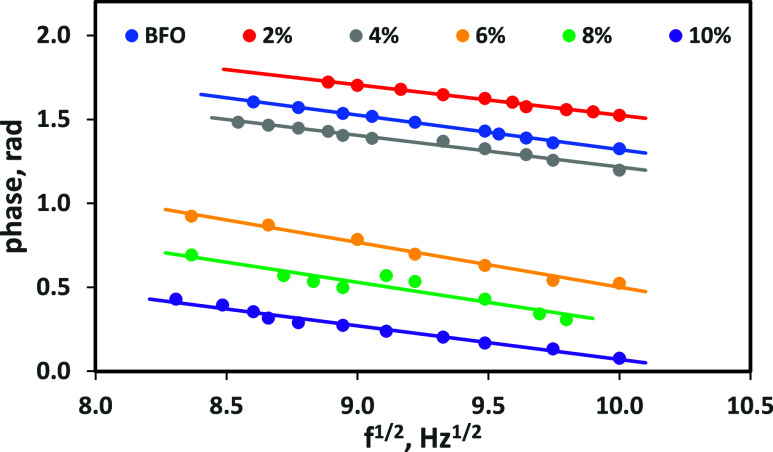
Phase of PPE signal for EB’s wavelength at 377 nm.

The samples’ thermal conductivities were determined by multiparameter fitting of theoretical curves obtained using the theoretical model (Appendix 1, Figure A1) to experimental data. The change in the BDS signal with doping level for different modulation frequencies of EB is presented on Argand’s graph (Figure 12). The thermal conductivity for undoped BFO was found to be 1.56 ± 0.06 W/m K. After doping the BFO material with 2% Pr and Co, the thermal conductivity was determined to be 1.73 ± 0.07 W/m·K. This indicates that introduction of a low level of dopant enhances thermal properties, which may be associated with superior crystalline organization and reduced defect density, as supported by the PXRD results, wherein the crystallite size increased from 58.17 nm in pure BFO to 66.57 nm in the 2% doped sample.^53,82^ A structure with less phonon-scattering centers is expected to facilitate more effective thermal conduction. After doping with higher concentrations of 4%, 6%, 8%, and 10%, the thermal conductivity follows a falling trend. The obtained values are 1.61 ± 0.06, 1.50 ± 0.06, 1.34 ± 0.05, and 1.29 ± 0.04 W/m·K, respectively. This decreasing trend results from introduction of larger structural distortions and phonon-scattering centers due to the formation of substitutional defects.^82^ The crystallite size obtained by PXRD analysis also shows a decreasing trend with increased dopant concentrations, even up to 24.42 nm for the 10% doped sample, which may be associated with an increased density of grain boundaries, contributing to thermal resistance. Additionally, morphological studies performed by SEM indicate that while lower doping initiates well-defined and less agglomerated grain morphology, higher doping levels lead to grain reagglomeration of particles and their nonhomogeneous distribution, which may influence heat transport through changes in particle distribution and connectivity.

**Figure 12:**
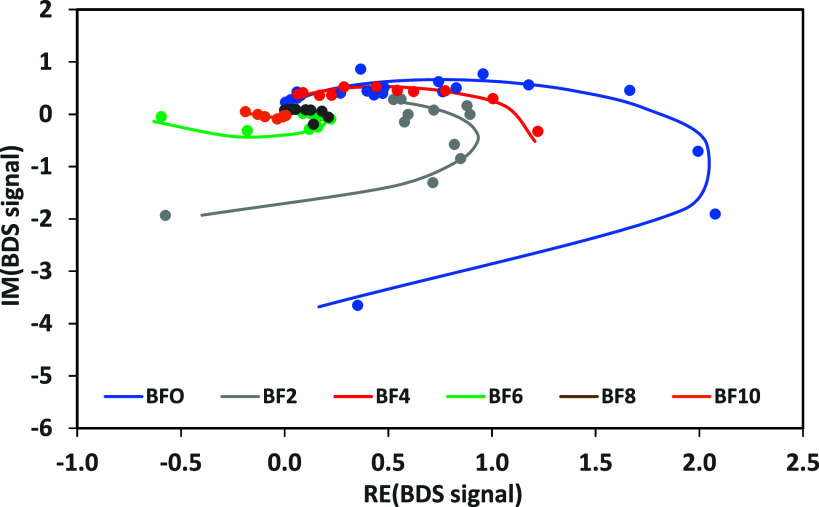
Thermal conductivity calculated using the excitation beam of wavelength 377 nm for the BFO and codoped samples.

Figure 13 shows a linear correlation between thermal conductivity (in W/m K) and thermal diffusivity (in mm^2^/s). This trend suggests that materials with higher thermal conductivity also exhibit higher thermal diffusivity across the series of doped and undoped BFO samples. This is consistent with the theoretical relationship between these two parameters, defined by the formula:

10 $$k=\alpha \rho C_{p}$$

where *k* and α are the thermal conductivity and diffusivity, respectively, ρ is the density, and *C_p_* is the specific heat capacity. Table 4 presents the measured thermal diffusivity and thermal conductivity values of the doped BFO samples under 377 nm excitation using the combined setup. The relative standard deviation (RSD) is less than 5%, which indicates good reliability of the method.

**Figure 13:**
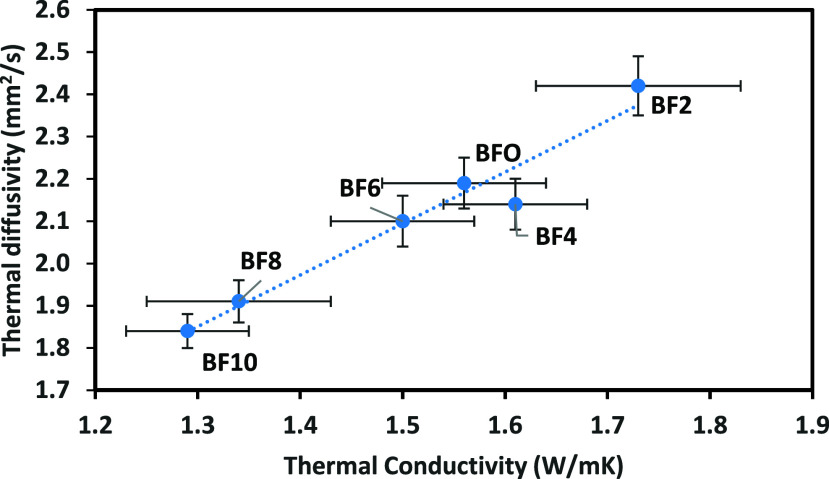
Correlation between thermal conductivity and thermal diffusivity of Pr–Co codoped BFO samples.

**Table 4:** Determined Thermal Diffusivity and Thermal Conductivity Values of Pure and Doped BFO Samples under 377 nm Excitation Using Combined BDS and PPE Setup

Samples	Thermal diffusivity (mm^2^/s)	Thermal conductivity (W/m·K)
BFO	2.19 ± 0.08	1.56 ± 0.06
BF2	2.42 ± 0.10	1.73 ± 0.07
BF4	2.14 ± 0.07	1.61 ± 0.06
BF6	2.10 ± 0.07	1.50 ± 0.06
BF8	1.91 ± 0.09	1.34 ± 0.05
BF10	1.84 ± 0.06	1.29 ± 0.04

In the next step, the band gap energy and carrier lifetime of the analyzed samples were determined. For this reason, the amplitudes and phases of the BDS signal as a function of EB modulation frequency were collected (Figure 14). The desired properties were obtained by performing multiparameter fittings of theoretical dependences (Appendix 2) to the experimental data (Figure 14). The thermal properties, determined previously, were taken now as constant values, whereas the band gap energy and carrier lifetime were the fitted parameters. The results are presented in Figures 15 and 16, respectively.

**Figure 14:**
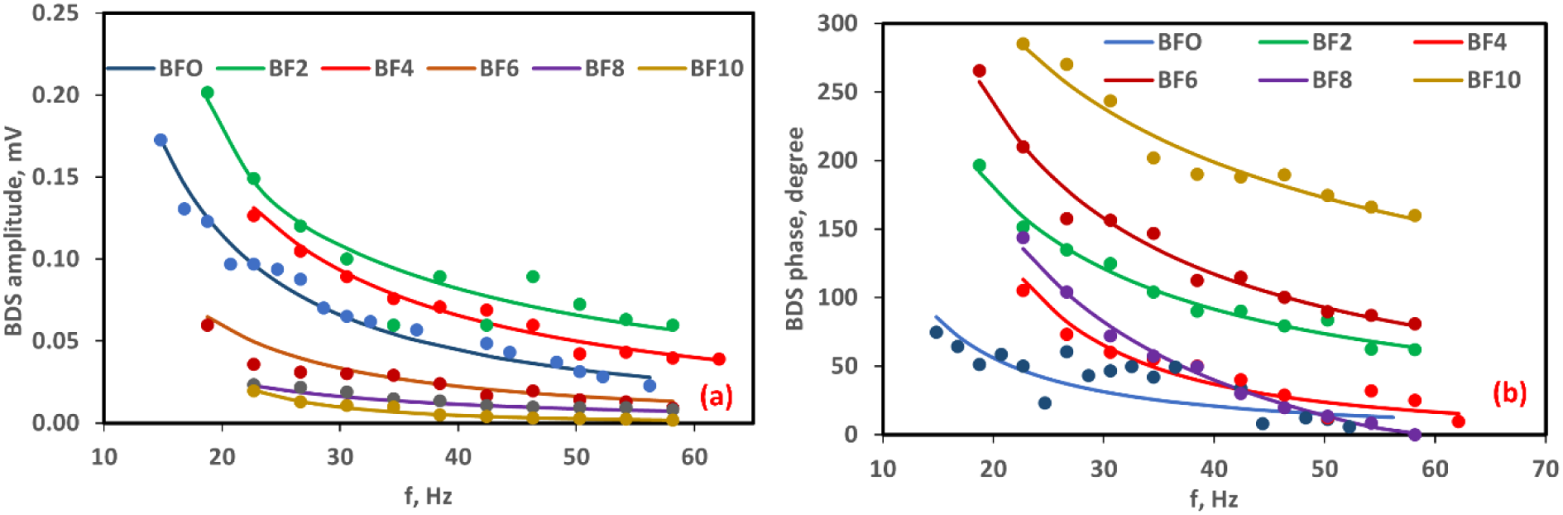
Amplitude (a) and phase (b) of BDS signal as a function of the EB modulation frequency.

**Figure 15:**
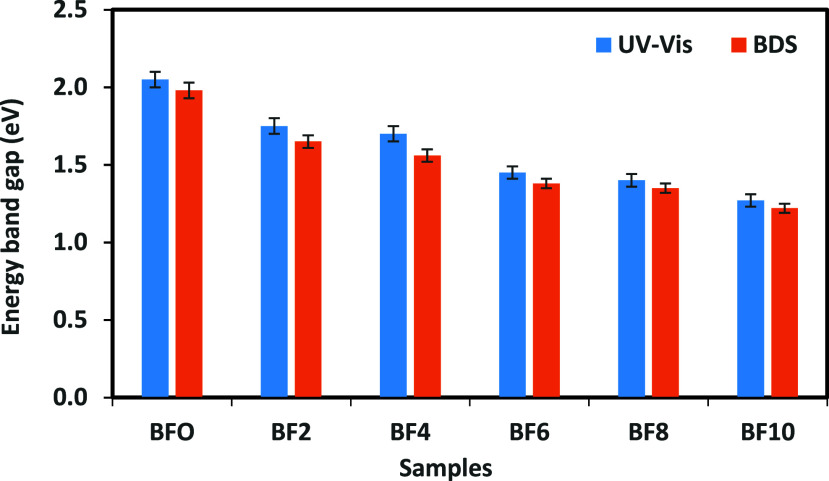
Variation in energy band gap determined by UV–vis and BDS for BFO and codoped BFO samples.

**Figure 16:**
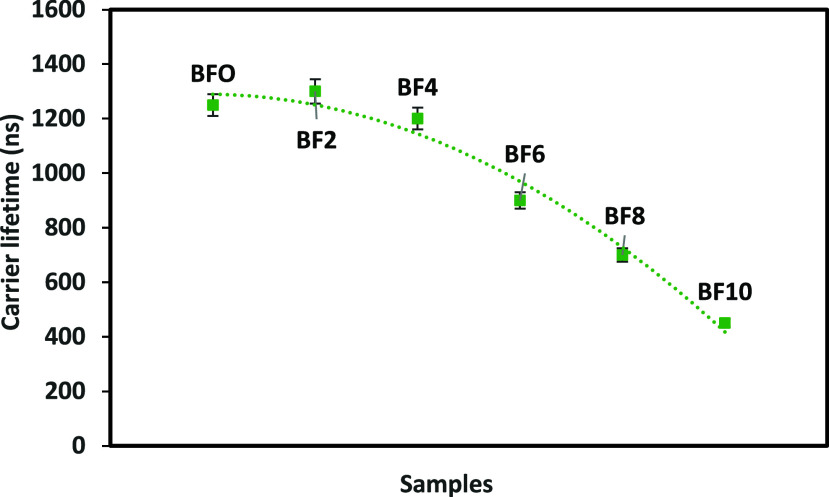
Variation in the carrier lifetime for pure and codoped BFO samples.

The band gap values determined by BDS and UV–vis techniques exhibit a consistent trend of gradual reduction with increasing dopant concentration. This qualitative agreement confirms that doping effectively modifies the optical properties of BFO-based materials. As the dopant concentration increases from 0% to 10%, the band gap shows a consistent decrease from 1.98 ± 0.05 eV to 1.22 ± 0.03 eV, which indicates that the absorption edge is gradually shifted toward longer wavelengths (red shift). This trend is similar to the trend observed in the band gap calculated by UV–vis. Determining the significance of observed changes in band gap values acquired by different procedures is how the qualitative analysis is carried out. For that reason, *p*-values were calculated at a 95% confidence level for three replicates of the procedure (Tukey’s test) as described in ref 83. If the computed *p*-value has a value of 0.05 or lower, the difference between compared properties is deemed statistically significant. No significant differences in the values of *E*_*g*_ are observed (*p*-value >0.05) in the case of all analyzed samples, though the physical basis of both techniques is different. UV–vis spectrophotometry is sensitive to light scattering unlike BDS. Furthermore, the BDS signal is the consequence of light energy conversion into heat. This process is determined by nonradiative intraband/interband transitions as well as nonradiative surface recombination^84^ and is not related to radiative transitions through the direct band gap that lead to emission of photons without heat generation. Thus, values of *E*_*g*_ determined by BDS are influenced by electron excitation through both direct and indirect band gaps and their nonradiative de-excitation through the indirect one, whereas in the case of UV–vis, the pure *E*_*g*_ is found. Since UV–vis spectrophotometry also indicates the presence of an indirect band in BFO materials, results obtained by both techniques coincide with each other.

The analysis of BDS measurements reveals also correlations between dopant concentration and carrier lifetime. The dependence of carrier lifetime on Pr–Co codoping concentration exhibits a nonmonotonic behavior. Initially, the carrier lifetime slightly increases at 2% doping (1300 ± 45 ns), indicating initial suppression of nonradiative recombination at low dopant levels. This enhancement can be associated with reduced defect-assisted recombination pathways and improved local structural ordering. However, with a further increase in doping concentration, the values of carrier lifetime drop significantly, reaching 450 ± 15 ns at a doping value of 10% (Figure 16). This reduction indicates an increase in recombination probability, which can be attributed to the introduction of defect states and enhanced lattice distortion at higher dopant concentrations.

A smaller band gap makes it easier for electrons to move from the valence band to the conduction band, which helps in the process of charge carrier generation and improves their movement.^20,60^ However, the significant reduction in carrier lifetime (1250 to 450 ns) at higher doping levels suggests that increased charge carrier recombination is accompanied by enhanced recombination pathways. A short carrier lifetime may limit the migration distance of electrons and holes, reducing their probability of participating in surface reactions.

Furthermore, the observed correlation between the values of carrier lifetimes and materials’ thermal properties^85,86^ shows strong defect sensitivity in the case of both carrier lifetime and thermal properties. Their values are affected by the presence and density of the introduced impurities. Pure BFO and samples with low dopant concentrations are expected to exhibit lower defect density, resulting in reduced phonon scattering and more efficient heat transport. In such systems, nonradiative recombination pathways are less prominent, leading to longer carrier lifetimes and improved charge carrier transport.

With increasing dopant concentration, the introduction of structural disorder and defect states enhances phonon scattering, thereby reducing the thermal transport properties. At the same time, these defect states can act as recombination centers, contributing to a reduction in carrier lifetime. Thus, both thermal transport and carrier lifetime exhibit sensitivity to the defect density.

The obtained values of carrier lifetimes coincide with the literature data, where carrier lifetimes in metal oxide semiconductors were found to be in a range from nanoseconds (ns) to milliseconds (ms).^87^ These values vary significantly depending on the material structure, doping concentration, and manufacturing quality. Introduction of dopants shortens these values significantly, making them fall in the pico- to nanosecond range.^88–90^

On the other hand, the thermal conductivity and diffusivity are the main factors that play a vital role in the material’s internal temperature distribution and its exchange with the surrounding. Increased thermal diffusivity enhances the fast process of heat distribution, while improved thermal conductivity supports the dissipation of localized heat generated during photocatalytic reactions. These effects may help in maintaining structural stability and limiting localized thermal accumulation within the material. In principle, efficient heat dissipation can influence charge carrier dynamics by reducing localized thermal fluctuations that may promote nonradiative recombination. However, this relationship is indirect, and thermal transport properties should be considered as contributing factors rather than dominant parameters controlling carrier lifetime or photocatalytic activity.^91–93^ Increased internal temperature inside the material can potentially accelerate the recombination process and affect structural stability. In other words, electrons may return to their valence band faster without participating in the photocatalytic reaction diminishing overall efficiency. Excessive heat potentially leads to lattice distortions, affecting the structural stability and electronic properties of the photocatalyst. This issue can be reduced by improving the thermal conductivity and thermal diffusivity of the material, which enables better dissipation of heat. In Pr–Co codoped BFO materials, a reduction of the band gap is generally noticed, which enhances visible-light absorption and promotes charge carrier generation. However, this reduction is generally accompanied by a reduction in charge carrier lifetime and thermal transport properties at higher doping levels, indicating increased defect density and recombination pathways. Notably, the thermal conductivity decreases with doping, except at 2% and 4% doping levels, where thermal conductivity increases. Thus, while codoping enhances the optical properties of BFO, it tends to adversely affect other crucial parameters, such as charge carrier lifetime and thermal transport. However, the 2% doped sample exhibits a favorable combination of thermal transport and carrier lifetime, whereas higher doping levels (e.g., BF10) achieve superior photocatalytic performance primarily due to enhanced light absorption. This indicates that the rate-limiting mechanism shifts with dopant concentration, reflecting the competing influence of thermal, optical, and electronic properties.

Therefore, the overall photocatalytic behavior arises from a balance between heat transport, charge carrier dynamics, and optical absorption rather than being governed by a single dominant factor.

### 3.7 Monitoring the Photocatalytic Reduction of Cr­(VI) to Cr­(III)

In the first step of the analysis, the photolability of the Cr–DPC complex was evaluated.^94^ For that purpose, 1 mg/L of Cr­(VI) in the Cr­(VI)–DPC complex was exposed to light. The Cr–DPC is a complex of purple color that absorbs light at a maximum wavelength of 540 nm. Under irradiation, the Cr–DPC solution’s color changes from purple to colorless. This is an indicator of its decomposition, which is also confirmed by the decrease in absorbance, as shown in Figure 17.

**Figure 17:**
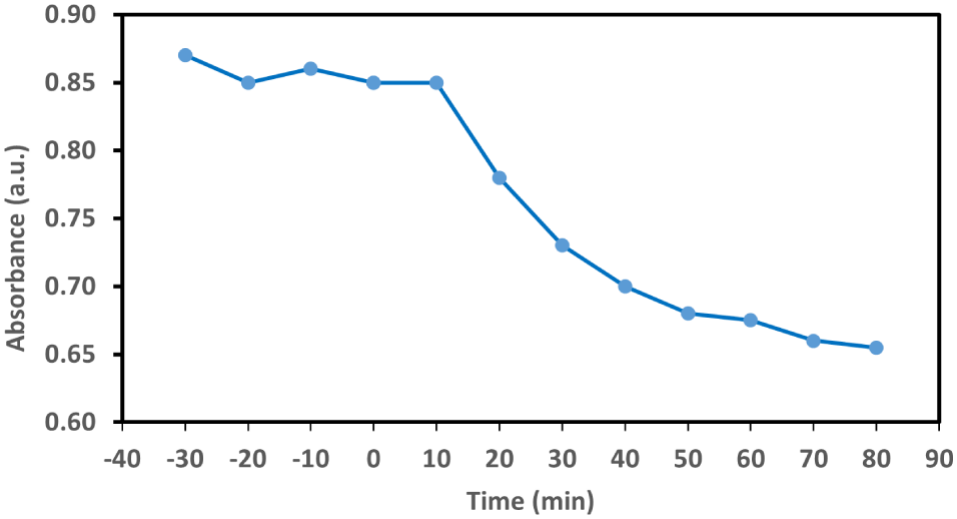
Absorbance as a function of time for the Cr–DPC complex under light illumination.

Although the Cr–DPC complex is sensitive to light and/or presents a tendency to degrade over time, it is stable enough for analysis since the drop in absorbance is less than 20% within 1.5 h after preparation. This suggests that the complex is stable within the time frame of the measurements; however, its potential influence under irradiation cannot be completely excluded. Therefore, the observed Cr­(VI) reduction is attributed to photocatalytic reduction under investigated conditions.

To monitor the photocatalytic reduction of Cr­(VI) to Cr­(III), UV–vis absorption measurements were taken in the wavelength range of 400–680 nm for various Cr­(VI) concentrations in the range between 0.1 and 1.0 mg/L upon reaction with DPC (Figure 17). To quantitatively estimate the Cr­(VI) concentration present in the solution during the photocatalytic process, the calibration curve was constructed (Figure 18).

**Figure 18:**
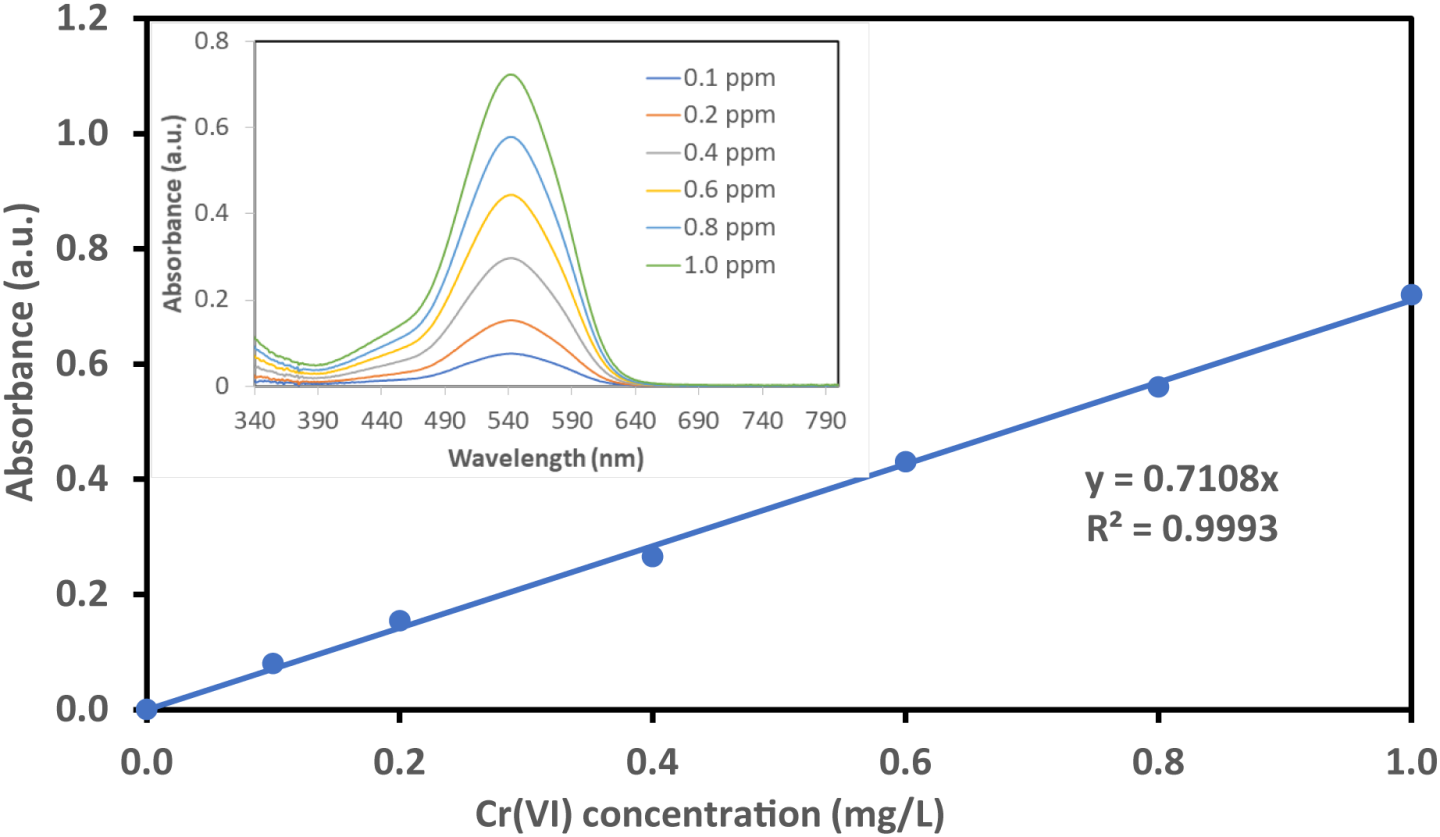
Calibration curve and related UV–vis spectra of the Cr–DPC complex for monitoring Cr­(VI) photocatalytic reduction.

The absorbance at 540 nm of the examined samples increases with an increase in Cr­(VI) amounts for the whole range of its concentration, according to Beer–Lambert’s law. Thus, the linearity of the method was obtained for the whole range of measured Cr­(VI) concentrations, with a correlation coefficient of *R* ^2^ = 0.995. This indicates good reliability of the analysis. The obtained LOD was found to be 0.03 mg/L, determining the lowest Cr­(VI) concentration that can be reliably detected.

The photocatalytic reduction efficiency of pure and Co–Pr codoped BFO photocatalysts was studied under sunlight irradiation by observing the temporal decrease in the absorbance of the Cr–DPC complex (Figure 19b). All of the examined materials exhibit a progressive reduction in absorbance with irradiation time, indicating their ability to facilitate the reduction process of Cr­(VI) to Cr­(III). The percentage decrease in absorbance of all samples was further recalculated to Cr­(VI) concentration change on the basis of calibration curve shown in Figure 18. These results reveal that BFO codoping with Co–Pr significantly enhances photocatalytic efficiency compared to the undoped BFO material. After 120 min of irradiation, the residual values of 1 mg/L Cr­(VI) initial concentration for BFO, BF2, BF4, BF6, BF8, and BF10 were 0.070, 0.058, 0.045, 0.052, 0.032, and 0.036 mg/L, respectively, which correspond to reduction efficiencies of 92.9%, 93.9%, 94.5%, 93.6%, 96.4%, and 96.8%. Although the Cr–DPC system without any photocatalysts exhibits some photoreduction (∼20%, Figure 17) in the presence of light, significantly enhanced reduction is observed in the presence of photocatalysts, which confirms their dominant role under the investigated conditions.

**Figure 19:**
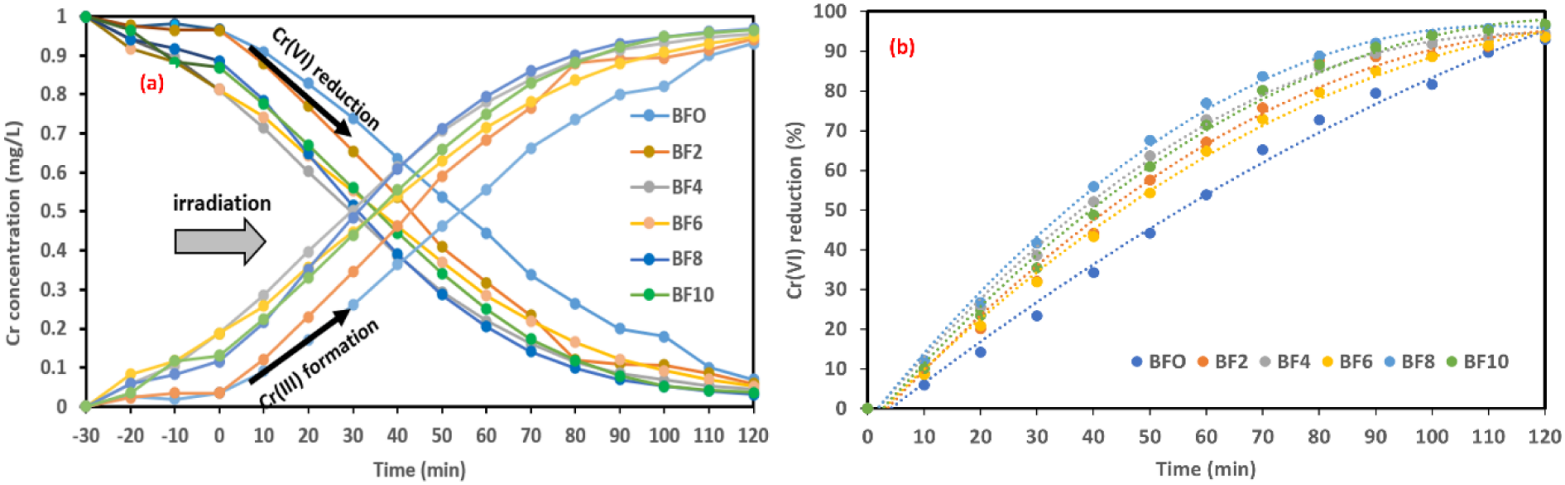
(a) Time-dependent photocatalytic process of Cr­(VI) reduction and Cr­(III) formation under sunlight irradiation. (b) Photocatalytic reduction efficiency (%) of Cr­(VI) as a function of irradiation time for pure BFO and Co–Pr codoped BFO catalysts.

Among these samples, BF8 and BF10 showed the highest and comparable photocatalytic efficiencies. This enhanced activity is primarily associated with improved visible light absorption due to band gap narrowing, which promotes charge carrier generation. At the same time, differences in carrier lifetime also influence the reduction process, as BF8 exhibits a relatively longer carrier lifetime compared with BF10. These observations indicate that photocatalytic performance arises from a balance between charge carrier generation and recombination dynamics rather than being governed by a single parameter.

It should also be noted that natural sunlight irradiation inherently involves minor intensity fluctuations; however, since all catalysts were evaluated under closely similar environmental conditions, the observed differences in photocatalytic performance can be reliably attributed to material properties rather than variation in irradiation. Despite the lack of absolute photon flux quantification, the consistency of trends across independently measured thermal, optical, and structural parameters supports the reliability of the comparative analysis.

The corresponding concentration of Cr­(III) was calculated based on the stoichiometric relation:

11 [Cr(III)]=Co−[Cr(VI)]

This assumes that each mole of reduced Cr (VI) is converted into one mole of Cr­(III), thereby maintaining the mass balance during the redox process. The concentration profiles of Cr­(VI) reduction and Cr­(III) formation during sunlight irradiation are shown in Figure 19a.

The procedure reproducibility was evaluated by calculating the reduction efficiency for three repetitions of photocatalytic cycling performed under similar weather conditions (see Section 2.2.4). The RSD of reduction efficiency was RSD <10%, which satisfies the EU criteria of ≤20%, showing good repeatability of the measurements. Furthermore, the reduction efficiency over three consecutive cycles of the photocatalytic process remains over 90%, which indicates high reusability and stability of the used photocatalysts.^95^

Among all of the photocatalysts, BF8 exhibits the fastest reduction rate of Cr­(VI), with residual concentrations falling below 0.03 mg/L after 120 min of exposure to solar light, while its Cr­(III) concentrations approached the initial Cr­(VI) value, confirming nearly complete reduction. Such a photocatalytic performance of BF8 arises from a favorable balance between reduced band gap, carrier lifetime, morphology, and thermal transport properties, rather than from any single dominant parameter.

To find its value, the plots of ln­(*C*/*C*_0_) versus irradiation time for pure and codoped BFO photocatalysts are drawn and presented in Figure 20. The slopes determine the values of the corresponding reduction rate constants *K* that can be found in Table 5. The dependences show linear behavior within the 30–100 min time frame of the analysis, validating the applicability of the pseudo-first-order model under these conditions. Generally, the value of *K* increases after introduction of dopants into pure BFO material, reaching its maximum value for BF8, which reflects the material’s enhanced photocatalytic activity. These results correlate well with the earlier obtained optical properties (narrowed band gaps, improved visible-light absorption, etc.).

**Figure 20:**
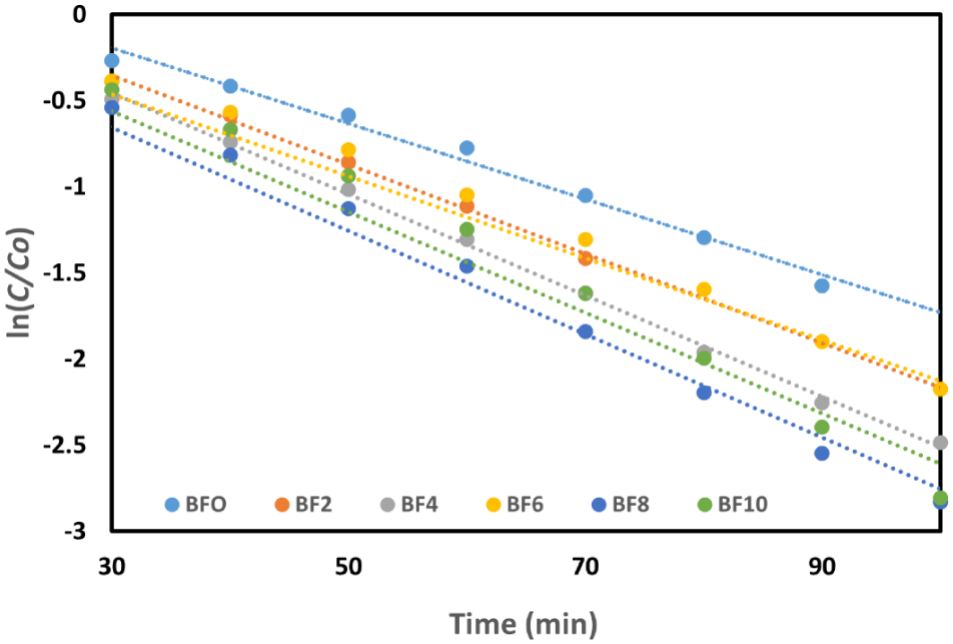
ln­(*C*/*C*_0_) dependences on irradiation time for pure and Co–Pr codoped BFO photocatalysts.

**Table 5:** Values of Photocatalytic Reduction Rates of Cr­(VI) Using BFO and Co–Pr Codoped BFO Catalysts

Samples	|K|, min^–1^
BFO	0.0220 ± 0.0009
BF2	0.0258 ± 0.0011
BF4	0.0292 ± 0.0012
BF6	0.0238 ± 0.0010
BF8	0.0300 ± 0.0013
BF10	0.0294 ± 0.0012

The improved photocatalytic performance of codoped samples is also consistent with modified optical and structural properties. Band gap narrowing enhances visible-light absorption, facilitating increased charge carrier generation. At the same time, variations in carrier lifetime and defect density influence recombination dynamics. Morphological changes, such as reduced agglomeration and improved particle distribution, may also contribute to an enhanced interaction between the catalyst surface and reactants.

BF8 exhibits the highest reduction rate constant (although it does not differ significantly from BF10, *p*-value >0.05), indicating more efficient reaction kinetics, while BF10 shows slightly higher overall reduction efficiency. Although BF10 has the narrowest band gap of all examined materials, it is characterized by a reduced charge carrier lifetime (to ∼450 ns), suggesting an increased recombination probability. Thus, photocatalytic performance in this system reflects a balance between charge carrier generation and recombination dynamics. Thus, no single property can be identified as the sole rate-limiting factor governing the reaction kinetics.

These observations highlight the importance of optimizing codopant concentration. Low-to-moderate Pr–Co codoping enhances photocatalytic activity through a synergistic combination of improved optical absorption and favorable charge carrier dynamics, whereas excessive doping introduces competing effects, such as increased recombination and reduced carrier lifetime.

The results are consistent with earlier reports on modified BFO materials, where different integration strategies enhance the photocatalytic reduction of Cr­(VI) by simultaneously improving light absorption and surface reactivity while controlling charge carrier recombination (Table 6).^96–98^

**Table 6:** Comparison of Performance Characteristics of BFO-Modified Photocatalysts Applied for Cr­(VI) Reduction

Modification	Band gap (eV)	Irradiation source	Efficiency (%)	Reference
Integration of carbon quantum dots	1.8–2.2	Artificial visible	63	^99,100^
Ca(II) doping	1.8–2.4		90	^101,102^
Mn(II) doping	1.3–1.5		91	^103^
Composite with CoFe_2_O_4_/Co_3_O_4_	2.0–2.8		99	^104^
Pr(III) doping	2.2		90	^29^
(CQD)–decoration	NA		75	^99^
Composite with Cl-*g*-C_3_N_4_	1.9		96	^105^
Composite with CoFe_2_O_4_/Co_3_O_4_	1.9		99	^104^
Co(III)/Pr(III)	1.3–1.7	Sunlight	96	This work

The results shown in Table 6 indicate that the studied materials exhibit competitive or superior reduction efficiencies under comparable or milder irradiation conditions.

## 4 Conclusion

In this work, a combined photothermal spectrometer integrating BDS and PPE systems was constructed and applied for multiparameter characterization of Pr–Co codoped BFO photocatalysts. The system enabled simultaneous determination of thermal, optical, and transport properties, providing a comprehensive understanding of material behavior. The results show that codoping significantly modifies the physiochemical properties of BFO. The results indicate reduction in band gap (from 1.98 ± 0.05 eV to 1.22 ± 0.03 eV) with increasing Pr and Co content (0–10%), while increasing dopant concentration led to reduced carrier lifetime (from ∼1300 ns to ∼450 ns) and decreased thermal transport due to increased defect density. The analysis of the photocatalytic reduction process of Cr­(VI) to Cr­(III) was found to achieve a reduction efficiency of up to ∼96% within 120 min of analysis under illumination with solar light, without the necessity of artificial light sources from moderate Pr–Co codoping (8%). Thus, it can be concluded that the optimal photocatalytic activity of Pr–Co codoped BFO photocatalysts is determined by a synergy between optical, thermal, and charge transport properties, rather than by maximization of any individual parameter.

Future investigations employing controlled light sources would allow quantitative determination of apparent quantum efficiency and further benchmarking of the photocatalytic performance.

## Figures and Tables

**A1 fig21:**
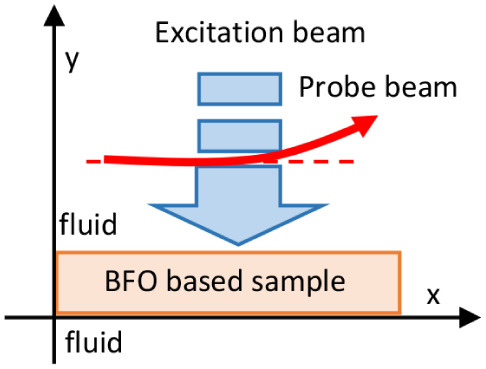
Geometry of the BDS experimental scheme used in the work.

## Data Availability

All the data can be provided upon request.

## Appendix 1

### A1. Theory

Both PPE and BDS techniques are based on the photothermal effect that is induced when a sample of interest absorbs electromagnetic radiation.^106,107^ As a result, the electrons in the atoms and molecules of the illuminated material are excited to higher energy levels. Due to non-radiative relaxation processes, the excited electrons return back to their ground states, releasing their energy in the form of heat without emitting photons. These processes involve collisions or internal vibrations within the sample’s molecules and/or its surroundings and occur very fast, thus leading to an increase in the local temperature of the sample and its surroundings. These temperature changes (thermal oscillations, TOs) carry information about sample’s thermo-optical properties (thermal diffusivity, conductivity, effusivity, absorption coefficient, energy band gap, etc.) and key characteristics that determine the sample’s internal heat conduction and external heat exchange (porosity, grain size, surface roughness, carrier lifetime, etc.).

### A1.1 PPE Calorimetry

In the case of the PPE technique, the TOs induced in the sample propagate to the pyroelectric sensor that converts them into an electrical signal through the pyroelectric effect. The slope of the PPE signal phase dependence on the square root of the EB modulation frequency can be used to determine the sample’s thermal diffusivity:^81^

A1
$$\phi_{PPE}=L_{s}{\left(\frac{\pi f}{D_{s}}\right)}^{1/2}$$

where *f* is the EB modulation frequency, *L*_*s*_ and *D*_*s*_ represent sample’s thickness and its thermal diffusivity, respectively.

### A1.2 BDS

In the case of BDS, a probe laser beam (PB) senses the TOs induced in the fluid layer over the sample’s surface.^108^ TOs create a refractive index gradient (RIG) and its changes (RIC), which change the PB intensity that is further measured by the quadrant photodiode (QPD):^53^

A2
$$S_{n}=K_{d}\left(\int_{0}^{+\infty }-\int_{-\infty }^{0}\right)dz_{D}\int_{-h}^{+\infty }dy_{D}I\left(x_{D},y_{D},z_{D}\right)$$

Here, *K*_*d*_ represents the detector constant, *h* is the PB height over the sample surface, and *I*(*x*_*D*_, *y*_*D*_, *z*_*D*_) is its intensity after undergoing TOs.

BDS measurements determine the signal’s amplitude and phase as a function of *f* changes. The sample’s thermal conductivity, band gap energy, and carrier lifetime are then determined by fitting the theoretical curves (eq A10, Appendix 2) to experimental data using the least squares method.

## Appendix 2

The geometry of the experimental setup used in the experiment is shown in Figure 21. The BFO-based sample has the form of a film with a thickness *L* that is in direct contact with a semi-infinite layer of fluid at both its interfaces.

It is assumed that the absorption of photons takes place throughout the entire sample thickness (the sample is thermally and optically thin). Furthermore, an assumption is made of the absorption in the fluid being much weaker than in the sample itself. The range of the modulation frequencies of TOs, used in the experiment, is chosen to ensure the thermal diffusion length μ being of the sample’s thickness size *L* or shorter to collect the information only from its volume. The heat fluctuations (TOs) induced in the sample and its surroundings are than found by the Fourier–Kirchhoff heat diffusion equation [^53^]­

A3
$$\frac{\partial v_{i}}{\partial t}=D_{Ti}[\nabla^{2}v_{i}+\frac{q_{i}}{k_{Ti}}]$$

where $v_{i}$ is the temperature oscillation, $D_{Ti}$ is the thermal diffusivity, $k_{Ti}$ is the thermal conductivity, and $q_{i}$ is the power density of the internal heat source. For EB much wider than the size of PB the induced TOs are 1D^112^. Thus, the heat source, generated by the absorption of the modulated EB is given by

A4
$$q_{si}\left(z,t\right)=\frac{1}{2}\gamma_{i}I_{EB}\exp\left[\overset{i-1}{\underset{m=1}{\sum }}\gamma_{m}\left(L_{m-1}-L_{m}\right)+\gamma_{i}\left(L_{i-1}-z\right)\right]\exp\left(2i\pi ft\right)$$

Here, $\gamma_{i}$ and $L_{i}$ are the optical absorption coefficient and thickness of the *i* ^*th*^ layer, respectively, $I_{EB}$ is the EB intensity, and *f* is the modulation frequency.

These TOs create refractive index gradients in the fluid above the sample, affecting the trajectory of a separate probe beam (PB). The deflection of the PB due to these gradients is expressed as:

A5
$$\Delta z\left(\varepsilon ,\tau \right)=n_{o}^{2}s_{T}\overset{\tau }{\underset{O}{\int }}\left(\tau -\tau^{\prime }\right)\frac{\partial v_{f}}{\partial z}d\tau^{\prime }$$

Here, $n_{o}$ is the refractive index of the undisturbed fluid, $s_{T}=\frac{1}{n_{o}}\left(\frac{dn}{dt}\right)$ is the temperature sensitivity of the refractive index, and τ is the complex path variable along the beam. This deflection causes the PB to diverge, altering its amplitude, approximated by:

A6
$$A\left(z\right)≅Ao\left(1+\Delta a\right),,Ao=Eo\frac{Z_{R}}{Z_{RC}},,Z_{R}=ka^{2}n_{o},,Z_{RC}=Z_{R}-iL_{o}$$

where a is the beam waist radius, Eo is the initial field amplitude, k is the wave number, and $L_{o}$ is the focal distance. In addition to amplitude change, the PB also undergoes a phase shift due to the same thermal effects, given by:

A7
$$\Delta \phi =kn_{o}^{2}s_{T}\overset{\tau }{\underset{O}{\int }}v_{f}\left[z\left(\tau^{\prime }\right)\right]d\tau^{\prime }$$

A8
$$\Delta \phi =\Delta \phi_{d}+\Delta \phi_{f}$$

where $\Delta \phi_{d}$ results from beam deflection and $\Delta \phi_{f}$ from changes in the refractive index.

These modifications in the probe beam affect its intensity at the detector placed at a position *z*_*D*_, which is expressed as:

A9
$$I\left(X_{D},Y_{D},Z_{D}\right)=I_{og}\left(X_{D},Y_{D,}Z_{D}\right)\left\{1-2kIm\left[\Delta \phi \left(Z_{D}\right)\right]+2Re\left[\Delta a\left(Z_{D}\right)\right]\right\}$$

with the undisturbed beam intensity described by:

A10
$$I_{0g}\left(X_{D},Y_{D},Z_{D}\right)={(E_{0}z_{D}Z_{RC}^{-1})}^{2}{|\exp\{ikn_{o}z_{D}[1-(x_{D}^{2}+y_{D}^{2}){(2z_{RC}^{2})}^{-1}{(1+iz_{D}z_{RC}^{-1})}^{-1}]\}|}^{2}$$

The final BDS signal is detected using a quadrant photodiode and is proportional to the integrated changes in PB amplitude and phase across the beam profile:

A11
$$S_{BDS}=2k_{d}\{\overset{+\infty }{\underset{0}{\int }}-\overset{0}{\underset{-z_{0}}{\int }}dx\overset{+\infty }{\underset{-\infty }{\int }}dy\left[Re\left(\Delta a\right)-kIm\left(\Delta \phi \right)\right]I_{o}\}$$

This signal varies sinusoidally with the modulation frequency of the EB, and in simplified form is given as:

A12
$$S_{BDS}=A_{BDS}\;\cos(2\pi ft+\mathrm{atan}(\theta_{mI}/\theta_{mR})+\phi_{BDS})$$

where *A*_*BDS*_ is the signal amplitude and $\phi_{BDS}$ is the phase shift resulting from thermal gradients and refractive index changes.

## Appendix 3

To determine the band gap energy and carrier lifetime, the theoretical model is modified to take into account the following processes that occur inside the examined material after its illumination by EB [112], resulting in creation of internal heat sources:

Bulk recombination:

A13
$$q_{sBR}\left(z,t\right)=E_{g}n\left(z,t\right)\tau^{-1}$$

• Surface recombination:

A14
$$q_{sSR}\left(z,t\right)=E_{g}\left[v_{SR0}\delta \left(z\right)+v_{SRL}\delta \left(z+L\right)\right]n\left(z,t\right)$$

• Intraband thermalization:

A15
$$q_{sIT}\left(z,t\right)=\alpha I_{0}{\left(h\nu \right)}^{-1}\left(h\nu -E_{g}\right)\exp\left[\alpha \left(z+L\right)+ift\right]$$

In eqs A13–A15, *E*_*g*_ denotes the energy band gap, *t* is the carrier lifetime, *n*(*z*,*t*) is the density of photogenerated carriers *v*_*sR0*_ and *v*_*sRL*_ are surface recombination velocities at surface *z* = 0 and *z* = −*L*, respectively, α is the optical absorption coefficient of the sample, and *I*_0_ is the intensity of EB.

A16
$$\frac{\partial n\left(z,t\right)}{\partial t}=D\frac{\partial^{2}n\left(z,t\right)}{\partial z^{2}}+g\left(z,t\right)-r\left(z,t\right)$$

A17
$$n\left(z,t\right)=\alpha I_{0}\tau {\left(2h\nu \right)}^{-1}\exp\left(\alpha z\right)$$

The carrier density *n*(*z*,*t*) found by solving the carrier diffusion equation:has the form:where *D* is the carrier diffusion coefficient, *g*(*z,t*) = *αI*_0_(2*h*ν)^−1^ exp­(α*z*) and *r*(*z,t*) = *n*(*z,t*)*τ* ^–1^ are generation and recombination rate of carriers, respectively

Finally, the total internal heat source in the thin film’s material can be written as

A18
$$q_{s}\left(z,t\right)=\alpha I_{0}E_{g}{\left(2h\nu \right)}^{-1}\left\{1+\tau \left[v_{SR0}\delta \left(z\right)+v_{SRL}\delta \left(z+L\right)\right]+2^{-1}\left[h\nu E_{g}^{-1}-1\right]\exp\left(\alpha L+ift\right)\right\}\exp\left(\alpha z\right)$$

The solution of the thermal diffusion equation (eq A1) in the fluid above the sample’s surface, with the internal heat source described by eq A6, gives the TOs that create RIG and RIC that lead to PB intensity changes, as described by eqs A3–A10.

The BDS signal carries than information about the thermal (thermal diffusivity *D*_*T*_, conductivity *k*_*T*_), optical (absorption coefficient α, energy band gap *E*_*g*_),^109^ and transport (minority carrier lifetime *t*, recombination velocity *v*_*r*_)^109^ parameters of the sample.
